# Navigating Nephrology's Decline Through a GPT-4 Analysis of Internal Medicine Specialties in the United States: Qualitative Study

**DOI:** 10.2196/57157

**Published:** 2024-10-10

**Authors:** Jing Miao, Charat Thongprayoon, Oscar Garcia Valencia, Iasmina M Craici, Wisit Cheungpasitporn

**Affiliations:** 1 Division of Nephrology and Hypertension, Department of Medicine, Mayo Clinic, 200 1st st sw, Rochester, MN, 55905, United States, 1 507 594 4700

**Keywords:** artificial intelligence, ChatGPT, nephrology fellowship training, fellowship matching, medical education, AI, nephrology, fellowship, United States, factor, chatbots, intellectual, complexity, work-life balance, procedural involvement, opportunity, career demand, financial compensation

## Abstract

**Background:**

The 2024 Nephrology fellowship match data show the declining interest in nephrology in the United States, with an 11% drop in candidates and a mere 66% (321/488) of positions filled.

**Objective:**

The study aims to discern the factors influencing this trend using ChatGPT, a leading chatbot model, for insights into the comparative appeal of nephrology versus other internal medicine specialties.

**Methods:**

Using the GPT-4 model, the study compared nephrology with 13 other internal medicine specialties, evaluating each on 7 criteria including intellectual complexity, work-life balance, procedural involvement, research opportunities, patient relationships, career demand, and financial compensation. Each criterion was assigned scores from 1 to 10, with the cumulative score determining the ranking. The approach included counteracting potential bias by instructing GPT-4 to favor other specialties over nephrology in reverse scenarios.

**Results:**

GPT-4 ranked nephrology only above sleep medicine. While nephrology scored higher than hospice and palliative medicine, it fell short in key criteria such as work-life balance, patient relationships, and career demand. When examining the percentage of filled positions in the 2024 appointment year match, nephrology’s filled rate was 66%, only higher than the 45% (155/348) filled rate of geriatric medicine. Nephrology’s score decreased by 4%‐14% in 5 criteria including intellectual challenge and complexity, procedural involvement, career opportunity and demand, research and academic opportunities, and financial compensation.

**Conclusions:**

ChatGPT does not favor nephrology over most internal medicine specialties, highlighting its diminishing appeal as a career choice. This trend raises significant concerns, especially considering the overall physician shortage, and prompts a reevaluation of factors affecting specialty choice among medical residents.

## Introduction

The National Resident Matching Program released the 2024 Nephrology fellowship match data on November 29, 2023 [1], revealing a significant downturn in the specialty’s appeal. Only 321 candidates secured nephrology positions, marking an 11% decrease from the prior year, leaving just more than half of the 180 nephrology programs filled. The trend is more obvious when considering that of the 488 spots available, a mere 66% (321/488) were taken [2], underscoring a persistent wane in the candidate-to-position ratio from 1.3 in 2011 to around 0.6 in recent years [3]. Alarmingly, only a small fraction of these roles were filled by US MD graduates, ranging from 15% to 26% between 2019 and 2024 [1,4].

This disinterest in nephrology is particularly concerning given the escalating shortage of nephrologists worldwide [5] and the burgeoning prevalence of chronic kidney conditions [6]. It is predicted that the United States alone may face a deficit of more than 139,000 physicians by 2030 [7], a scenario that casts a long shadow over the future of nephrology care and its sustainability. The publication of annual match data consistently amplifies these worries, leading to persistent debates [3,8-11]. Nonetheless, the underlying causes of this critical issue are still largely unexamined.

In this context, there is a growing curiosity about the role of advanced artificial intelligence (AI) tools such as ChatGPT in reshaping medical education and practice [12-15]. This study uses ChatGPT to analyze and juxtapose nephrology with other internal medicine specialties, aiming to illuminate the influences shaping medical career choices today and provide insights into decision-making in the evolving landscape of medical career planning.

## Methods

### Specialties Examined in This Study

Within the realm of internal medicine, there are 17 fellowship specialties other than Nephrology [4]. There are 4 advanced fellowships such as Adult Congenital Heart Disease, Advanced Heart Failure & Transplant Cardiology, Clinical Cardiac Electrophysiology, and Interventional Pulmonology, which are typically not options for Internal Medicine residents or internists who might consider a nephrology fellowship. Hence, these 4 advanced specialties were not included in our study.

### Study Design

GPT-4, a sophisticated iteration of ChatGPT, was prompted to provide insights into choosing between nephrology and other 13 internal medicine specialties. To examine that the ChatGPT’s response did not depend on the sequence of fellowships presented in the query, we also asked ChatGPT to choose between other specialties and nephrology in reverse scenarios.

The prompts used in this study have been provided in (Multimedia Appendix 1) and are presented in screenshot format. Specifically, we asked:

*If you need to choose nephrology or [insert specialty name] fellowship, which one do you choose, you can describe but at the end you need choose one; each aspect comparisons may choose scores of 1‐10*.

For the reverse scenarios, we used:

*If you need to choose [insert specialty name] or nephrology fellowship, which one do you choose, you can describe but at the end you need choose one; each aspect comparisons may choose scores of 1‐10*.

ChatGPT’s responses are also presented in screenshot format. To prevent our content from being used to train the models, we disabled the “Data controls—Improve the model for everyone” option in the setting of ChatGPT. To minimize potential biases from the AI’s memory of prior interactions and ensure the independence of each prompt and response, we started each query in a new chat session.

### Evaluation

In evaluating the decline in interest in Nephrology, we used 7 criteria identified independently by ChatGPT. These criteria include (1) intellectual challenge and complexity, (2) work-life balance, (3) procedural involvement, (4) research and academic opportunities, (5) patient relationships and continuity of care, (6) career opportunity and demand, and (7) financial compensation. These factors were chosen based on their relevance and applicability to fellowship selection in the real world. While these criteria are consistent with those used in the analysis of other specialties, it is important to note that they were not established by the authors themselves.

Each criterion was rated on a scale from 1 to 10. We did not train ChatGPT on how to score each criterion, such as defining what constitutes a 1/10 or a 9/10. We did not use any weighting anchors in the ChatGPT scoring process.

We calculated a cumulative score for each specialty based on the 7 criteria, resulting in a maximum possible score of 70 per specialty. The comparative ratio of nephrology’s score in each criterion over other specialties was calculated. Nephrology’s score in each criterion was also compared with the average score of all other specialties.

### Ethical Considerations

This study does not include human participants (no human subjects experimentation or intervention was conducted) and so does not require institutional review board approval.

## Results

**Figure 1.**
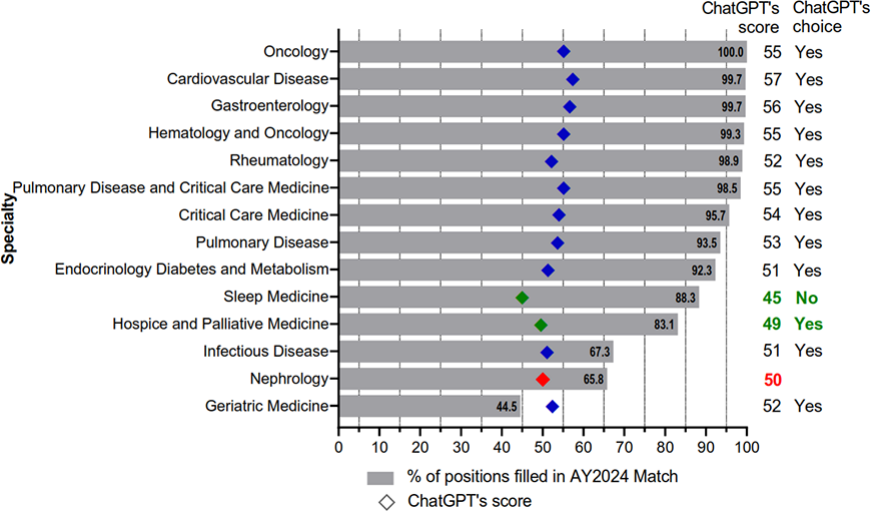
ChatGPT’s score and the fellowship position fill rates. Seven criteria for each specialty were assessed by ChatGPT. Each criterion was scored on a scale from 1 to 10, resulting in a maximum possible score of 70. The total score assigned by ChatGPT to each specialty, along with its fellowship recommendations, is presented using diamonds. Nephrology’s score ( red diamond) surpassed only those of sleep medicine and hospice and palliative medicine (green diamond). ChatGPT recommended nephrology as a fellowship option only when compared with sleep medicine. ChatGPT’s score and choice mainly align with the rank of positions filled in 2024 reported by the National Resident Matching Program (gray bar). The fill rate for nephrology fellowships (321/488, 65.8%) was only higher than that of geriatric medicine (155/348, 44.5%). AY: appointment year.

ChatGPT favored only nephrology over a single specialty, sleep medicine (Table 1). Despite accruing a total score surpassing that of hospice and palliative medicine, ChatGPT opted for palliative medicine instead (Figure 1). Analysis of the 2024 appointment year match data revealed that 66% of nephrology positions were filled, a rate that exceeded only that of geriatric medicine, which stood at 45% (155/348) (Figure 1).

**Figure 2.**
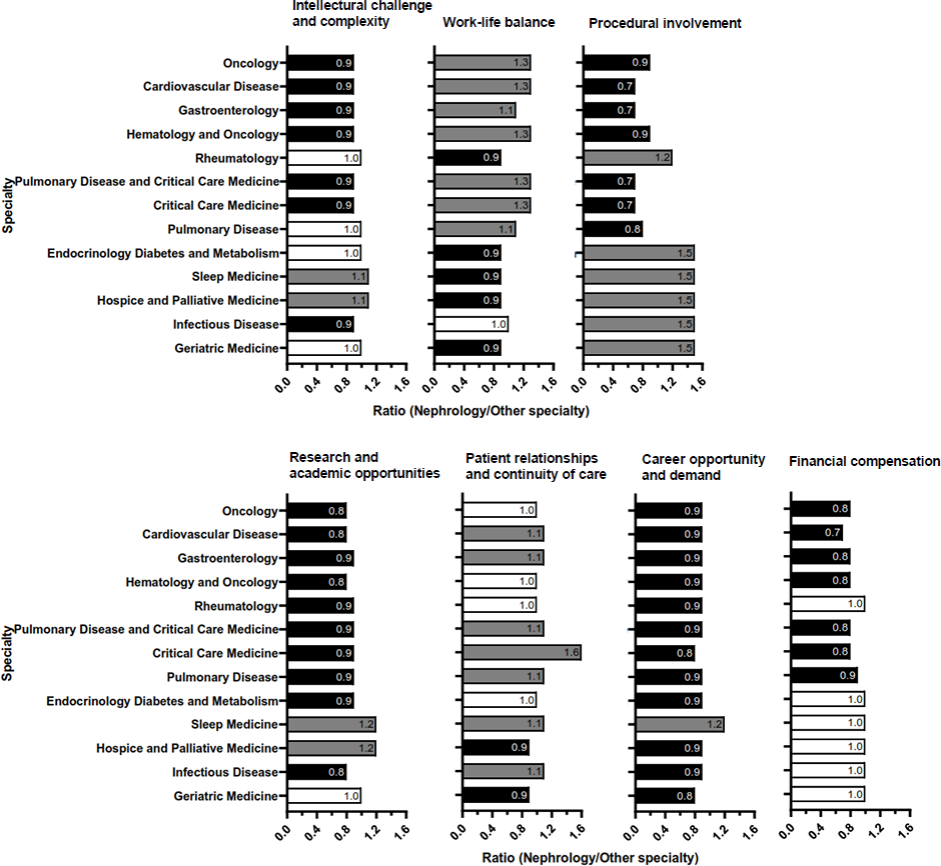
Comparisons of nephrology with other specialties in each criterion. The comparative ratio of nephrology to other specialties in terms of 7 criteria. color label for the ratio of nephrology to other specialties: black <1, white=1, and gray>1.

**Figure 3.**
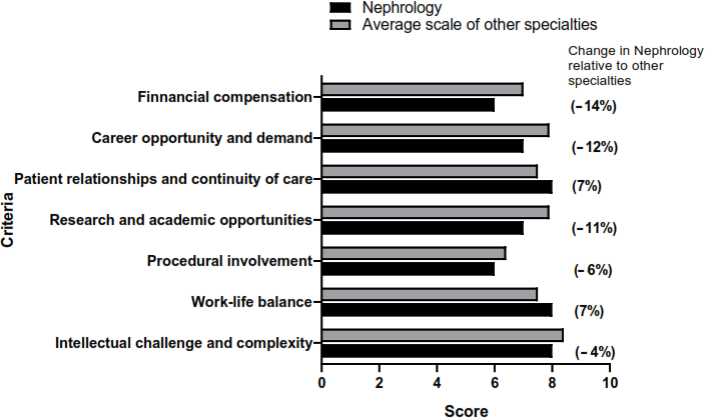
Comparisons of nephrology with the average score of other specialties in each criterion. Comparisons of the scale of various criteria in nephrology against the average scale for the other 13 specialties. Change in nephrology relative to other specialties shows the reduction in the percentage for nephrology relative to the average scale of the other 13 specialties.

Upon examining specific parameters, nephrology ranked comparatively lower in terms of career demand, research opportunities, and financial remuneration than most other specialties (Figure 2). Specifically, nephrology experienced a decline ranging from 4% to 14% in 5 principal domains: intellectual challenge, procedural involvement, career demand, research prospects, and financial compensation. Nonetheless, nephrology exhibited a relative improvement, with a 7% increase noted in both the aspects of work-life balance and the development of patient relationships (Figure 3).

The same scores and choices were observed when we asked GPT-4 to evaluate other specialties over nephrology in reverse scenarios (Multimedia Appendix 1).

**Table 1.** Scale of nephrology and 13 other specialties on 7 criteria.

	Intellectual complexity	Work-life balance	Procedural involvement	Research and academic opportunities	Patient relationships and continuity of care	Career opportunity and demand	Financial compensation	Total score	ChatGPT’s choice over Nephrology^a^
Nephrology	8	8	6	7	8	7	6	50	N/A^b^
Geriatric medicine	8	9	4	7	9	9	6	52	Yes
Infectious disease	9	8	4	9	7	8	6	51	Yes
Hospice and palliative medicine	7	9	4	6	9	8	6	49	Yes
Sleep medicine	7	9	4	6	7	6	6	45	No
Endocrinology, diabetes, and metabolism	8	9	4	8	8	8	6	51	Yes
Pulmonary Disease	8	7	8	8	7	8	7	53	Yes
Critical care medicine	9	6	9	8	5	9	8	54	Yes
Pulmonary disease and critical care medicine	9	6	9	8	7	8	8	55	Yes
Rheumatology	8	9	5	8	8	8	6	52	Yes
Hematology and oncology	9	6	7	9	8	8	8	55	Yes
Gastroenterology	9	7	9	8	7	8	8	56	Yes
Cardiovascular disease	9	6	9	9	7	8	9	57	Yes
Oncology	9	6	7	9	8	8	8	55	Yes
Average score^c^	8.5	7.2	6.8	8.0	7.5	7.9	7.2	52.7	N/A

a ChatGPT’s preference for Nephrology compared with other Internal Medicine specialties when it comes to fellowship selection.

b N/A: not applicable.

c The average score of individual criterion across all other 13 specialties.

## Discussion

### Principal Findings

The results underscore a diminished appeal in selecting nephrology as a career path, evident even in the preferences of sophisticated AI models such as ChatGPT when simulating fellowship choices. The factors contributing to this waning interest are likely diverse and personal. However, the significance of this trend cannot be overlooked, particularly in the context of the prevailing physician shortage.

Notably, the nephrology fellowship achieved a total score of 50, marginally surpassing hospice and palliative medicine, which scored 49, and sleep medicine at 45, among the 13 internal medicine specialties. However, ChatGPT recommended only nephrology over sleep medicine. Despite nephrology’s higher overall score, ChatGPT favored hospice and palliative medicine due to its superior work-life balance, patient relationships, and career demand—especially pertinent given the aging population and increasing need for quality end-of-life care (Textbox 1). Nevertheless, ChatGPT noted that nephrology might be more suitable for those with a preference for technical aspects such as fluid and electrolyte management and renal pathophysiology. The lower score in intellectual complexity and procedural involvement for hospice and palliative medicine may have also influenced ChatGPT’s decision.

In fact, the reality for nephrologists is increasingly challenging. They face a growing workload and a diminishing control over their schedules, a situation exacerbated by the rising incidence of kidney diseases, especially chronic kidney disease and end-stage kidney disease [6]. The demands of the profession are extensive, involving long outpatient waitlists, demanding inpatient services, unpredictable night calls, and frequent visits to multiple dialysis units. These responsibilities, particularly the travel between units, consume significant time and effort [16]. In addition to their clinical duties, the 2023 Medscape Nephrologist Compensation Report states that nephrologists need to devote an average of 18.1 hours per week to support tasks such as paperwork and administration [17].

In terms of intellectual rigor, nephrology is on par with other specialties (8 vs 8.5). However, a national survey among internal medicine residents revealed that the field’s broad scope and the complexity of kidney-related pathologies and physiologies deter many potential entrants [18-20]. The patients under nephrological care often present some of the most medically complex cases, marked by a plethora of comorbidities, intricate medication regimes, and a higher mortality risk. Despite these challenges, some find the diverse clinical conditions and the vast scope of practice in nephrology appealing [21,22]. Studies indicate that intellectual curiosity about kidney-related issues is a primary motivator for some choosing this career path [22]. Factors influencing this choice include a passion for the subject, a favorable work-life balance, mentorship availability, and exposure to the field [23]. However, exposure to nephrology during medical training is limited, with only a minority experiencing a rotation in this specialty during their clinical years, compared with a higher percentage during residency [18]. This limited exposure might be due to the complex nature of renal care, a relative scarcity of hands-on procedures compared with other specialties, and a lack of visible role models or mentors [24]. These factors contribute to the lower proportion of US MD graduates pursuing nephrology [4], highlighting a significant gap in early medical education and potential areas for enhancement in the field’s approach to attracting and nurturing future talent.

Nephrology lags behind other medical specialties in financial reward, demand, and research prospects, impacting its appeal. It shows a 16% lower preference score in financial compensation, underscoring concerns highlighted in the 2023 Medscape Nephrologist Compensation Report. Nephrologists’ average annual income falls below the median for all specialties [17]. In addition, the report indicates that nephrologists are in the bottom third of all specialties regarding how often they feel fairly compensated for their talents and time. In last year’s report, nephrologists were ranked in the bottom spot. Furthermore, a survey among internal medicine residents reveals that the main obstacles deterring them from nephrology also include perceived financial inadequacy, intellectual rigor, work-life balance, and the potential to positively influence patient outcomes [18]. We recognize that financial considerations are multifaceted and significantly influenced by regional differences. Factors such as salary potential, cost of living, educational costs, and regional demand play crucial roles in deciding to choose nephrology as a specialty. Our study aimed to provide a general perspective, but we acknowledge the critical impact of regional financial factors on this decision-making process.

Recently, the perception of limited advancements and new therapeutic developments in nephrology has been recognized as a significant deterrent for choosing nephrology among internal medicine residents in the United States [25]. Despite ChatGPT scoring nephrology’s career demand lower than the average for other specialties, there is, in reality, an increasing demand in the field of nephrology. This rise is observed not just in terms of patient needs but also in career opportunities within the specialty. This increase is driven by several factors, including the rising prevalence of chronic kidney disease, aging populations, and the associated complexities in managing these conditions [6]. As the number of individuals requiring specialized kidney care escalates, so does the need for skilled nephrologists to provide comprehensive and effective treatment. This surge in patient demand is creating more career opportunities within nephrology, indicating a promising future for those entering the field.

To address nephrology fellowship underfill rates, the American Society of Nephrology implemented measures such as the All-In policy, STARS (Students and Residents), and TREKS (Tutored Research Education for Kidney Scholars) programs [3,10]. ChatGPT suggests promoting nephrology’s significance; enhancing training; fostering research; incorporating technology; encouraging collaboration, mentorship, and career growth; advocating work-life balance; increasing awareness; ensuring competitive pay; and broadening subspecialty choices.

Our study has certain constraints. It is important to acknowledge that the criteria for ranking established by ChatGPT are not fully transparent. While the criteria seem reasonable and applicable to real-world fellowship selection, the exact methodology and rationale behind their selection remain partially opaque. The overall score differences between nephrology and the 13 other specialties are relatively small. This limitation should be considered when interpreting the findings of our study. In addition, we did not use weighting anchors in the ChatGPT scoring process, recognizing that the importance of each criterion may vary. To mitigate hallucinations, a major concern in ChatGPT’s responses, we implemented several measures such as asking the same questions in reverse scenarios, preventing the content from being used to train the models, and starting each query in a new chat session to minimize potential biases from the AI’s memory of prior interactions. While these measures reduce the likelihood of hallucinations, we cannot completely exclude their possibility. It remains uncertain whether ChatGPT’s choices truly reflect residents’ sentiments. Addressing whether this issue originates from biases in perception or from broader systemic problems is crucial. Moreover, ChatGPT’s scores and choices were consistent when the same questions were presented in reverse scenarios. However, previous studies, including our own [26], have shown varying levels of repeatability in ChatGPT’s responses, indicating that repeatability might depend on the nature and type of the question. Finally, although we obtained consistent results using another ChatGPT account with the same prompts, we cannot entirely exclude the possibility that different ChatGPT accounts might produce varying results due to differences in settings, usage habits, repeatability, and other unknown factors. Further research is needed to address this issue.

Textbox 1. **ChatGPT’s conclusion on deciding to pursue a fellowship in Hospice and Palliative Medicine.**

Conclusion Based on this analysis, I would recommend choosing a Hospice and Palliative Medicine fellowship. It scores higher in work-life balance, patient relationships, and career demand, particularly relevant in the context of an aging population and the increasing need for quality end-of-life care. This choice should align with personal interests and career goals, especially if one is drawn to patient-centered, holistic care. If a preference lies in more technical aspects like fluid and electrolyte management and renal pathophysiology, then Nephrology might be more suitable.

### Conclusions

ChatGPT, as an AI model, shows no bias toward nephrology over other internal medicine branches in fellowship choices, highlighting a broader decline in interest for this specialty. This trend is driven by factors such as financial incentives, career demands, and opportunities for research, which significantly influence specialty decisions. Moreover, intellectual stimulation and work-life balance are key factors. This issue, whether due to perceived or real barriers, demands immediate action in light of the physician shortage. Addressing these deterrents is essential to boost nephrology’s attractiveness and fulfill the increasing demand for nephrologists, thereby maintaining exemplary health care standards.

## Supplementary material

10.2196/57157 Multimedia Appendix 1 ChatGPT’s responses to fellowship selection between nephrology and other 13 internal medicine specialties as well as between other specialties and nephrology in reverse scenarios.

## Abbreviations

AI: artificial intelligence
