# Supplementary material for: Navigating Nephrology's Decline Through a GPT-4 Analysis of Internal Medicine Specialties in the United States: Qualitative Study
Source: JMIR Med Educ. 2024 Oct 10;10:e57157. doi: 10.2196/57157 (PMC11486450; doi:10.2196/57157)
Supplement: Multimedia Appendix 1 [file mededu-v10-e57157-s001.pdf]

Supplementary Data ChatGPT's responses to fellowship selection between Nephrology and other thirteen Internal Medicine specialties as well as between other specialties and Nephrology in reverse scenarios.

Nephrology vs Critical Care Medicine

You

If you need to choose nephrology or critical care medicine fellowship, which one do you choose, you can describe but at the end you need choose one; each aspect comparisons may choose scores of 1-10

ChatGPT

Choosing between a Nephrology and a Critical Care Medicine fellowship involves a multifaceted analysis, considering various aspects such as intellectual stimulation, work-life balance, procedural involvement, and future career opportunities. I will score these aspects from 1 to 10 for each specialty and conclude with a recommendation based on the analysis.

1. **Intellectual Stimulation and Complexity**:
   - **Nephrology**: This field offers deep insight into complex fluid, electrolyte, and acid-base balance, and requires a strong understanding of physiology and pathophysiology. It's intellectually demanding and constantly evolving. Score: 8/10.
   - **Critical Care Medicine**: This specialty demands a broad knowledge base, quick decision-making, and the ability to manage critically ill patients with multi-organ issues. It's intellectually challenging and dynamic. Score: 9/10.
2. **Work-Life Balance**:
   - **Nephrology**: Generally offers a more predictable schedule compared to critical care, with fewer night shifts. It allows for a better work-life balance in most settings. Score: 8/10.
   - **Critical Care Medicine**: Often involves shift work with irregular hours, including nights and weekends, which can be more challenging for work-life balance. Score: 6/10.

3. **Procedural Involvement**:
   - **Nephrology**: Involves procedures like kidney biopsies and dialysis catheter placements, but to a lesser extent than some other specialties. Score: 6/10.
   - **Critical Care Medicine**: High in procedural intensity, including intubations, central line placements, and management of mechanical ventilation. Score: 9/10.
4. **Research and Academic Opportunities**:
   - **Nephrology**: Offers substantial opportunities in both clinical and basic research, particularly in areas like dialysis, transplant, and hypertension. Score: 7/10.
   - **Critical Care Medicine**: Also rich in research opportunities, especially in areas like sepsis, ARDS, and mechanical ventilation. Score: 8/10.
5. **Patient Relationships and Continuity of Care**:
   - **Nephrology**: Allows for long-term patient relationships, especially in chronic kidney disease management and dialysis. Score: 8/10.
   - **Critical Care Medicine**: Typically involves acute, short-term relationships with patients and families in crisis situations. Score: 5/10.
6. **Career Opportunities and Demand**:
   - **Nephrology**: Steady demand, particularly given the aging population and prevalence of conditions like hypertension and diabetes. Score: 7/10.
   - **Critical Care Medicine**: High demand, especially in underserved areas and in the context of pandemics or widespread health emergencies. Score: 9/10.
7. **Financial Compensation**:
   - **Nephrology**: Generally lower compared to some other specialties, but still offers a comfortable living. Score: 6/10.
   - **Critical Care Medicine**: Typically offers higher compensation due to the intensity and demands of the work. Score: 8/10.

Conclusion: Based on this analysis, if I were to choose, I would recommend **Critical Care Medicine**. It scores higher in intellectual stimulation, procedural involvement, research opportunities, career demand, and financial compensation. However, this choice is contingent on one's personal preferences and career aspirations, particularly valuing high-intensity, acute care environments over long-term patient relationships and a more predictable schedule.

You

If you need to choose Critical Care Medicine or Nephrology fellowship, which one do you choose, you can describe but at the end you need choose one; each aspect comparisons may choose scores of 1-10.

ChatGPT

In deciding between a Critical Care Medicine and a Nephrology fellowship, we will assess various aspects such as intellectual challenge, work-life balance, procedural involvement, research opportunities, patient relationships, career demand, and financial compensation. Each aspect will be scored from 1 to 10 for both specialties, leading to a final recommendation.

1. **Intellectual Challenge and Complexity**:
   - **Critical Care Medicine**: Demands a broad knowledge base, quick decision-making, and the ability to manage critically ill patients with multi-organ issues. It's intellectually challenging and dynamic. Score: 9/10.
   - **Nephrology**: Offers deep insight into complex fluid, electrolyte, and acid-base balance, requiring a strong understanding of physiology and pathophysiology. It's intellectually demanding and constantly evolving. Score: 8/10.
2. **Work-Life Balance**:
   - **Critical Care Medicine**: Often involves shift work with irregular hours, including nights and weekends, which can be more challenging for work-life balance. Score: 6/10.
   - **Nephrology**: Generally offers a more predictable schedule compared to critical care, with fewer night shifts. It allows for a better work-life balance in most settings. Score: 8/10.

3. **Procedural Involvement**:
   - **Critical Care Medicine**: High in procedural intensity, including intubations, central line placements, and management of mechanical ventilation. Score: 9/10.
   - **Nephrology**: Involves procedures like kidney biopsies and dialysis catheter placements, but to a lesser extent than some other specialties. Score: 6/10.
4. **Research and Academic Opportunities**:
   - **Critical Care Medicine**: Also rich in research opportunities, especially in areas like sepsis, ARDS, and mechanical ventilation. Score: 8/10.
   - **Nephrology**: Offers substantial opportunities in both clinical and basic research, particularly in areas like dialysis, transplant, and hypertension. Score: 7/10.
5. **Patient Relationships and Continuity of Care**:
   - **Critical Care Medicine**: Typically involves acute, short-term relationships with patients and families in crisis situations. Score: 5/10.
   - **Nephrology**: Allows for long-term patient relationships, especially in chronic kidney disease management and dialysis. Score: 8/10.
6. **Career Opportunities and Demand**:
   - **Critical Care Medicine**: High demand, especially in underserved areas and in the context of pandemics or widespread health emergencies. Score: 9/10.
   - **Nephrology**: Steady demand, particularly given the aging population and prevalence of conditions like hypertension and diabetes. Score: 7/10.
7. **Financial Compensation**:
   - **Critical Care Medicine**: Typically offers higher compensation due to the intensity and demands of the work. Score: 8/10.
   - **Nephrology**: Generally lower compared to some other specialties, but still offers a comfortable living. Score: 6/10.

Conclusion: Based on this analysis, I would recommend choosing a **Critical Care Medicine** fellowship. It scores higher in intellectual stimulation, procedural involvement, research opportunities, career demand, and financial compensation. This choice should align with personal preferences and career aspirations, particularly if one values high-intensity, acute care environments over long-term patient relationships and a more predictable schedule.

You

If you need to choose nephrology or infectious disease fellowship, which one do you choose, you can describe but at the end you need choose one; each aspect comparisons may choose scores of 1-10

ChatGPT

In deciding between a Nephrology and an Infectious Disease fellowship, we will evaluate various aspects such as intellectual challenge, work-life balance, procedural involvement, research opportunities, patient relationships, career demand, and financial compensation. Each aspect will be scored from 1 to 10 for both specialties, culminating in a final recommendation.

1. **Intellectual Challenge and Complexity**:
   - **Nephrology**: Offers a deep understanding of complex fluid, electrolyte, and acid-base balance. It's intellectually demanding with a strong focus on physiology and pathophysiology. Score: 8/10.
   - **Infectious Disease**: Involves a broad and evolving knowledge base, dealing with a wide range of pathogens and clinical presentations. It's intellectually stimulating and requires staying current with emerging diseases and treatments. Score: 9/10.
2. **Work-Life Balance**:
   - **Nephrology**: Generally provides a more predictable schedule with fewer emergencies, allowing for a better work-life balance. Score: 8/10.
   - **Infectious Disease**: Also offers a relatively balanced lifestyle with fewer night shifts, although outbreaks and pandemics can temporarily increase workload. Score: 8/10.

3. **Procedural Involvement**:
   - **Nephrology**: Involves some procedures like kidney biopsies and dialysis catheter placements. Score: 6/10.
   - **Infectious Disease**: Less procedural compared to other specialties, focusing more on diagnostic and therapeutic decision-making. Score: 4/10.
4. **Research and Academic Opportunities**:
   - **Nephrology**: Offers opportunities in clinical and basic research, particularly in areas like dialysis and transplant. Score: 7/10.
   - **Infectious Disease**: Rich in research opportunities, especially with emerging infections, antibiotic resistance, and vaccine development. Score: 9/10.
5. **Patient Relationships and Continuity of Care**:
   - **Nephrology**: Allows for long-term patient relationships in chronic disease management. Score: 8/10.
   - **Infectious Disease**: Often involves acute care but can also include long-term management of chronic infections like HIV. Score: 7/10.
6. **Career Opportunities and Demand**:
   - **Nephrology**: Steady demand due to aging populations and increasing prevalence of conditions like hypertension and diabetes. Score: 7/10.
   - **Infectious Disease**: High demand, especially in the context of global health challenges and emerging infections. Score: 8/10.
7. **Financial Compensation**:
   - **Nephrology**: Generally offers a comfortable living, though not among the highest-paying specialties. Score: 6/10.
   - **Infectious Disease**: Similar to Nephrology in terms of compensation, not typically among the highest earners. Score: 6/10.

Conclusion: Based on this analysis, I would recommend choosing an **Infectious Disease** fellowship. It scores higher in intellectual challenge, research opportunities, and career demand, particularly relevant in the context of global health challenges. This choice, however, should align with personal interests, especially if one has a strong inclination towards managing chronic conditions and performing procedural tasks, in which case Nephrology would be more suitable.

Infectious Disease vs Nephrology

You

If you need to choose infectious disease or nephrology fellowship, which one do you choose, you can describe but at the end you need choose one; each aspect comparisons may choose scores of 1-10

ChatGPT

In choosing between an Infectious Disease and a Nephrology fellowship, we will evaluate various aspects such as intellectual challenge, work-life balance, procedural involvement, research opportunities, patient relationships, career demand, and financial compensation. Each aspect will be scored from 1 to 10 for both specialties, leading to a final recommendation.

1. **Intellectual Challenge and Complexity**:
   - **Infectious Disease**: Involves a broad and evolving knowledge base, dealing with a wide range of pathogens and clinical presentations. It's intellectually stimulating and requires staying current with emerging diseases and treatments. Score: 9/10.
   - **Nephrology**: Offers a deep understanding of complex fluid, electrolyte, and acid-base balance. It's intellectually demanding with a strong focus on physiology and pathophysiology. Score: 8/10.
2. **Work-Life Balance**:
   - **Infectious Disease**: Offers a relatively balanced lifestyle with fewer night shifts, although outbreaks and pandemics can temporarily increase workload. Score: 8/10.
   - **Nephrology**: Generally provides a more predictable schedule with fewer emergencies, allowing for a better work-life balance. Score: 8/10.

3. **Procedural Involvement**:
   - **Infectious Disease**: Less procedural compared to other specialties, focusing more on diagnostic and therapeutic decision-making. Score: 4/10.
   - **Nephrology**: Involves some procedures like kidney biopsies and dialysis catheter placements. Score: 6/10.
4. **Research and Academic Opportunities**:
   - **Infectious Disease**: Rich in research opportunities, especially with emerging infections, antibiotic resistance, and vaccine development. Score: 9/10.
   - **Nephrology**: Offers opportunities in clinical and basic research, particularly in areas like dialysis and transplant. Score: 7/10.
5. **Patient Relationships and Continuity of Care**:
   - **Infectious Disease**: Often involves acute care but can also include long-term management of chronic infections like HIV. Score: 7/10.
   - **Nephrology**: Allows for long-term patient relationships in chronic disease management. Score: 8/10.
6. **Career Opportunities and Demand**:
   - **Infectious Disease**: High demand, especially in the context of global health challenges and emerging infections. Score: 8/10.
   - **Nephrology**: Steady demand due to aging populations and increasing prevalence of conditions like hypertension and diabetes. Score: 7/10.
7. **Financial Compensation**:
   - **Infectious Disease**: Similar to Nephrology in terms of compensation, not typically among the highest earners. Score: 6/10.
   - **Nephrology**: Generally offers a comfortable living, though not among the highest-paying specialties. Score: 6/10.

Conclusion: Based on this analysis, I would recommend choosing an **Infectious Disease** fellowship. It scores higher in intellectual challenge, research opportunities, and career demand, particularly relevant in the context of global health challenges. This choice should align with personal interests and career goals, especially if one has a strong inclination towards managing chronic conditions and performing procedural tasks, in which case Nephrology might be more suitable.

You

If you need to choose nephrology or cardiology fellowship, which one do you choose, you can describe but at the end you need choose one; each aspect comparisons may choose scores of 1-10

ChatGPT

In deciding between a Nephrology and a Cardiology fellowship, we will evaluate various aspects such as intellectual challenge, work-life balance, procedural involvement, research opportunities, patient relationships, career demand, and financial compensation. Each aspect will be scored from 1 to 10 for both specialties, culminating in a final recommendation.

1. **Intellectual Challenge and Complexity**:
   - **Nephrology**: Involves complex fluid, electrolyte, and acid-base balance understanding. It's intellectually demanding with a strong focus on physiology and pathophysiology. Score: 8/10.
   - **Cardiology**: Requires mastery over a wide range of cardiac conditions, interventions, and evolving technologies. It's highly intellectually stimulating and challenging. Score: 9/10.
2. **Work-Life Balance**:
   - **Nephrology**: Generally offers a more predictable schedule with fewer emergencies, allowing for a better work-life balance. Score: 8/10.
   - **Cardiology**: Often involves on-call duties and emergency interventions, which can be more challenging for work-life balance. Score: 6/10.

3. **Procedural Involvement**:
   - **Nephrology**: Involves procedures like kidney biopsies and dialysis catheter placements, but less procedural than cardiology. Score: 6/10.
   - **Cardiology**: High in procedural intensity, including catheterizations, angioplasties, and electrophysiology procedures. Score: 9/10.
4. **Research and Academic Opportunities**:
   - **Nephrology**: Offers opportunities in clinical and basic research, particularly in areas like dialysis and transplant. Score: 7/10.
   - **Cardiology**: Rich in research opportunities, with rapid advancements in areas like interventional cardiology, heart failure, and electrophysiology. Score: 9/10.
5. **Patient Relationships and Continuity of Care**:
   - **Nephrology**: Allows for long-term patient relationships in chronic disease management. Score: 8/10.
   - **Cardiology**: Also allows for long-term relationships, especially in chronic conditions like heart failure, but can also involve acute care. Score: 7/10.
6. **Career Opportunities and Demand**:
   - **Nephrology**: Steady demand due to aging populations and increasing prevalence of conditions like hypertension and diabetes. Score: 7/10.
   - **Cardiology**: High demand, given the prevalence of cardiac diseases. Score: 8/10.
7. **Financial Compensation**:
   - **Nephrology**: Offers a comfortable living, though not among the highest-paying specialties. Score: 6/10.
   - **Cardiology**: Typically among the higher-paying specialties due to procedural and clinical demand. Score: 9/10.

Conclusion: Based on this analysis, I would recommend choosing a **Cardiology** fellowship. It scores higher in intellectual challenge, procedural involvement, research opportunities, career demand, and financial compensation. This choice should align with personal interests and career goals, especially if one has a strong inclination towards high-intensity, procedure-oriented environments. If long-term patient management and a more predictable schedule are preferred, then Nephrology might be more suitable.

Cardiology vs Nephrology

You

If you need to choose cardiology or nephrology fellowship, which one do you choose, you can describe but at the end you need choose one; each aspect comparisons may choose scores of 1-10

ChatGPT

Choosing between a Cardiology and a Nephrology fellowship involves evaluating various aspects such as intellectual challenge, work-life balance, procedural involvement, research opportunities, patient relationships, career demand, and financial compensation. Each aspect will be scored from 1 to 10 for both specialties, leading to a final recommendation.

1. **Intellectual Challenge and Complexity**:
   - **Cardiology**: Involves a wide range of cardiac conditions, interventions, and evolving technologies. It's highly intellectually stimulating and challenging. Score: 9/10.
   - **Nephrology**: Offers deep insight into complex fluid, electrolyte, and acid-base balance, requiring a strong understanding of physiology and pathophysiology. It's intellectually demanding and constantly evolving. Score: 8/10.
2. **Work-Life Balance**:
   - **Cardiology**: Often involves on-call duties and emergency interventions, which can be more challenging for work-life balance. Score: 6/10.
   - **Nephrology**: Generally offers a more predictable schedule compared to cardiology, with fewer night shifts. It allows for a better work-life balance in most settings. Score: 8/10.

3. **Procedural Involvement**:
    - **Cardiology**: High in procedural intensity, including catheterizations, angioplasties, and electrophysiology procedures. Score: 9/10.
    - **Nephrology**: Involves some procedures like kidney biopsies and dialysis catheter placements, but less procedural than cardiology. Score: 6/10.
4. **Research and Academic Opportunities**:
    - **Cardiology**: Rich in research opportunities, with rapid advancements in areas like interventional cardiology, heart failure, and electrophysiology. Score: 9/10.
    - **Nephrology**: Offers opportunities in clinical and basic research, particularly in areas like dialysis and transplant. Score: 7/10.
5. **Patient Relationships and Continuity of Care**:
    - **Cardiology**: Also allows for long-term relationships, especially in chronic conditions like heart failure, but can also involve acute care. Score: 7/10.
    - **Nephrology**: Allows for long-term patient relationships in chronic kidney disease management and dialysis. Score: 8/10.
6. **Career Opportunities and Demand**:
    - **Cardiology**: High demand, given the prevalence of cardiac diseases. Score: 8/10.
    - **Nephrology**: Steady demand due to aging populations and increasing prevalence of conditions like hypertension and diabetes. Score: 7/10.
7. **Financial Compensation**:
    - **Cardiology**: Typically among the higher-paying specialties due to procedural and clinical demand. Score: 9/10.
    - **Nephrology**: Generally offers a comfortable living, though not among the highest-paying specialties. Score: 6/10.

Conclusion: Based on this analysis, I would recommend choosing a **Cardiology** fellowship. It scores higher in intellectual challenge, procedural involvement, research opportunities, career demand, and financial compensation. However, this choice should align with personal preferences and career aspirations, particularly valuing high-intensity, procedure-oriented environments. If long-term patient management and a more predictable schedule are preferred, then Nephrology might be more suitable.

Nephrology vs Endocrinology, Diabetes, and Metabolism

You

If you need to choose Nephrology or Endocrinology, Diabetes, and Metabolism fellowship, which one do you choose, you can describe but at the end you need choose one; each aspect comparisons may choose scores of 1-10.

ChatGPT

In choosing between a Nephrology and an Endocrinology, Diabetes, and Metabolism fellowship, we will evaluate various aspects such as intellectual challenge, work-life balance, procedural involvement, research opportunities, patient relationships, career demand, and financial compensation. Each aspect will be scored from 1 to 10 for both specialties, culminating in a final recommendation.

1. **Intellectual Challenge and Complexity**:
   - **Nephrology**: Offers deep insight into complex fluid, electrolyte, and acid-base balance, requiring a strong understanding of physiology and pathophysiology. It's intellectually demanding and constantly evolving. Score: 8/10.
   - **Endocrinology, Diabetes, and Metabolism**: Involves a broad spectrum of disorders, requiring deep knowledge in hormonal balance, metabolic diseases, and diabetes management. It's intellectually stimulating and complex. Score: 8/10.
2. **Work-Life Balance**:
   - **Nephrology**: Generally offers a more predictable schedule compared to some other specialties, with fewer night shifts. It allows for a better work-life balance in most settings. Score: 8/10.
   - **Endocrinology, Diabetes, and Metabolism**: Also known for a favorable work-life balance, with fewer emergencies and a more regular schedule. Score: 9/10.
3. **Procedural Involvement**:
   - **Nephrology**: Involves procedures like kidney biopsies and dialysis catheter placements, but to a lesser extent than some other specialties. Score: 6/10.
   - **Endocrinology, Diabetes, and Metabolism**: Less procedural than Nephrology, focusing more on diagnostic and therapeutic decision-making rather than invasive procedures. Score: 4/10.

4. **Research and Academic Opportunities**:
   - **Nephrology**: Offers substantial opportunities in both clinical and basic research, particularly in areas like dialysis, transplant, and hypertension. Score: 7/10.
   - **Endocrinology, Diabetes, and Metabolism**: Rich in research opportunities, especially in diabetes management, thyroid disorders, and metabolic diseases. Score: 8/10.
5. **Patient Relationships and Continuity of Care**:
   - **Nephrology**: Allows for long-term patient relationships, especially in chronic kidney disease management and dialysis. Score: 8/10.
   - **Endocrinology, Diabetes, and Metabolism**: Also offers long-term patient relationships, particularly in managing chronic conditions like diabetes and thyroid disorders. Score: 8/10.
6. **Career Opportunities and Demand**:
   - **Nephrology**: Steady demand, particularly given the aging population and prevalence of conditions like hypertension and diabetes. Score: 7/10.
   - **Endocrinology, Diabetes, and Metabolism**: High demand, especially with the increasing prevalence of diabetes and obesity. Score: 8/10.

7. **Financial Compensation**:
   - **Nephrology**: Generally lower compared to some other specialties, but still offers a comfortable living. Score: 6/10.
   - **Endocrinology, Diabetes, and Metabolism**: Comparable to Nephrology in terms of compensation, not among the highest-paying specialties but offers a stable income. Score: 6/10.

Conclusion: Based on this analysis, I would recommend choosing an **Endocrinology, Diabetes, and Metabolism** fellowship. It scores slightly higher in work-life balance, research opportunities, and career demand, particularly relevant in the context of the growing prevalence of metabolic diseases. This choice should align with personal interests and career goals, especially if one is drawn to managing chronic metabolic and hormonal disorders. If a preference lies in fluid and electrolyte management and renal pathophysiology, then Nephrology might be more suitable.

Endocrinology, Diabetes, and Metabolism vs Nephrology

You

If you need to choose Endocrinology, Diabetes, and Metabolism or Nephrology fellowship, which one do you choose, you can describe but at the end you need choose one; each aspect comparisons may choose scores of 1-10.

ChatGPT

In deciding between an Endocrinology, Diabetes, and Metabolism and a Nephrology fellowship, we will assess various aspects such as intellectual challenge, work-life balance, procedural involvement, research opportunities, patient relationships, career demand, and financial compensation. Each aspect will be scored from 1 to 10 for both specialties, leading to a final recommendation.

1. **Intellectual Challenge and Complexity**:
   - **Endocrinology, Diabetes, and Metabolism**: Involves a broad spectrum of disorders, requiring deep knowledge in hormonal balance, metabolic diseases, and diabetes management. It's intellectually stimulating and complex. Score: 8/10.
   - **Nephrology**: Offers deep insight into complex fluid, electrolyte, and acid-base balance, requiring a strong understanding of physiology and pathophysiology. It's intellectually demanding and constantly evolving. Score: 8/10.
2. **Work-Life Balance**:
   - **Endocrinology, Diabetes, and Metabolism**: Known for a favorable work-life balance, with fewer emergencies and a more regular schedule. Score: 9/10.
   - **Nephrology**: Generally offers a more predictable schedule compared to some other specialties, with fewer night shifts. It allows for a better work-life balance in most settings. Score: 8/10.
3. **Procedural Involvement**:
   - **Endocrinology, Diabetes, and Metabolism**: Less procedural than Nephrology, focusing more on diagnostic and therapeutic decision-making rather than invasive procedures. Score: 4/10.
   - **Nephrology**: Involves procedures like kidney biopsies and dialysis catheter placements, but to a lesser extent than some other specialties. Score: 6/10.

4. **Research and Academic Opportunities**:
   - **Endocrinology, Diabetes, and Metabolism**: Rich in research opportunities, especially in diabetes management, thyroid disorders, and metabolic diseases. Score: 8/10.
   - **Nephrology**: Offers substantial opportunities in both clinical and basic research, particularly in areas like dialysis, transplant, and hypertension. Score: 7/10.
5. **Patient Relationships and Continuity of Care**:
   - **Endocrinology, Diabetes, and Metabolism**: Offers long-term patient relationships, particularly in managing chronic conditions like diabetes and thyroid disorders. Score: 8/10.
   - **Nephrology**: Allows for long-term patient relationships, especially in chronic kidney disease management and dialysis. Score: 8/10.
6. **Career Opportunities and Demand**:
   - **Endocrinology, Diabetes, and Metabolism**: High demand, especially with the increasing prevalence of diabetes and obesity. Score: 8/10.
   - **Nephrology**: Steady demand, particularly given the aging population and prevalence of conditions like hypertension and diabetes. Score: 7/10.

7. **Financial Compensation**:
   - **Endocrinology, Diabetes, and Metabolism**: Comparable to Nephrology in terms of compensation, not among the highest-paying specialties but offers a stable income. Score: 6/10.
   - **Nephrology**: Generally lower compared to some other specialties, but still offers a comfortable living. Score: 6/10.

Conclusion: Based on this analysis, I would recommend choosing an **Endocrinology, Diabetes, and Metabolism** fellowship. It scores slightly higher in work-life balance, research opportunities, and career demand, particularly relevant in the context of the growing prevalence of metabolic diseases. This choice should align with personal interests and career goals, especially if one is drawn to managing chronic metabolic and hormonal disorders. If a preference lies in fluid and electrolyte management and renal pathophysiology, then Nephrology might be more suitable.

Nephrology vs Gastroenterology

You

If you need to choose Nephrology or Gastroenterology fellowship, which one do you choose, you can describe but at the end you need choose one; each aspect comparisons may choose scores of 1-10.

ChatGPT

In choosing between a Nephrology and a Gastroenterology fellowship, we will evaluate various aspects such as intellectual challenge, work-life balance, procedural involvement, research opportunities, patient relationships, career demand, and financial compensation. Each aspect will be scored from 1 to 10 for both specialties, culminating in a final recommendation.

1. **Intellectual Challenge and Complexity**:
   - **Nephrology**: Offers deep insight into complex fluid, electrolyte, and acid-base balance, requiring a strong understanding of physiology and pathophysiology. It's intellectually demanding and constantly evolving. Score: 8/10.
   - **Gastroenterology**: Involves a broad spectrum of diseases affecting the GI tract, liver, and pancreas. It requires a deep understanding of a diverse range of pathologies and procedures. Score: 9/10.
2. **Work-Life Balance**:
   - **Nephrology**: Generally offers a more predictable schedule compared to some other specialties, with fewer night shifts. It allows for a better work-life balance in most settings. Score: 8/10.
   - **Gastroenterology**: Can have variable schedules with on-call duties and procedures, which might affect work-life balance. Score: 7/10.

3. **Procedural Involvement**:
   - **Nephrology**: Involves procedures like kidney biopsies and dialysis catheter placements, but to a lesser extent than Gastroenterology. Score: 6/10.
   - **Gastroenterology**: Highly procedural, involving endoscopies, colonoscopies, and more advanced procedures like ERCP. Score: 9/10.

4. **Research and Academic Opportunities**:
   - **Nephrology**: Offers substantial opportunities in both clinical and basic research, particularly in areas like dialysis, transplant, and hypertension. Score: 7/10.
   - **Gastroenterology**: Also rich in research opportunities, especially in areas like inflammatory bowel disease, hepatology, and advanced endoscopic techniques. Score: 8/10.
5. **Patient Relationships and Continuity of Care**:
   - **Nephrology**: Allows for long-term patient relationships, especially in chronic kidney disease management and dialysis. Score: 8/10.
   - **Gastroenterology**: Offers a mix of acute and chronic patient care, with opportunities for long-term relationships in chronic GI and liver diseases. Score: 7/10.
6. **Career Opportunities and Demand**:
   - **Nephrology**: Steady demand, particularly given the aging population and prevalence of conditions like hypertension and diabetes. Score: 7/10.
   - **Gastroenterology**: High demand, especially with the growing awareness of GI and liver diseases and the need for cancer screening. Score: 8/10.
7. **Financial Compensation**:
   - **Nephrology**: Generally lower compared to some other specialties, but still offers a comfortable living. Score: 6/10.
   - **Gastroenterology**: Typically offers high[illegible] compensation due to procedural work and high demand. Score: 8/10.

Conclusion: Based on this analysis, I would recommend choosing a **Gastroenterology** fellowship. It scores higher in intellectual challenge, procedural involvement, research opportunities, career demand, and financial compensation. This choice should align with personal interests and career goals, especially if one is drawn to a diverse, procedure-intensive field with a mix of acute and chronic patient care. If a preference lies in fluid and electrolyte management and renal pathophysiology, then Nephrology might be more suitable.

Gastroenterology vs Nephrology

You

If you need to choose Gastroenterology or Nephrology fellowship, which one do you choose, you can describe but at the end you need choose one; each aspect comparisons may choose scores of 1-10.

ChatGPT

In deciding between a Gastroenterology and a Nephrology fellowship, we will assess various aspects such as intellectual challenge, work-life balance, procedural involvement, research opportunities, patient relationships, career demand, and financial compensation. Each aspect will be scored from 1 to 10 for both specialties, leading to a final recommendation.

1. **Intellectual Challenge and Complexity**:
   - **Gastroenterology**: Involves a broad spectrum of diseases affecting the GI tract, liver, and pancreas. It requires a deep understanding of a diverse range of pathologies and procedures. Score: 9/10.
   - **Nephrology**: Offers deep insight into complex fluid, electrolyte, and acid-base balance, requiring a strong understanding of physiology and pathophysiology. It's intellectually demanding and constantly evolving. Score: 8/10.
2. **Work-Life Balance**:
   - **Gastroenterology**: Can have variable schedules with on-call duties and procedures, which might affect work-life balance. Score: 7/10.
   - **Nephrology**: Generally offers a more predictable schedule compared to some other specialties, with fewer night shifts. It allows for a better work-life balance in most settings. Score: 8/10.
3. **Procedural Involvement**:
   - **Gastroenterology**: Highly procedural, involving endoscopies, colonoscopies, and more advanced procedures like ERCP. Score: 9/10.
   - **Nephrology**: Involves procedures like kidney biopsies and dialysis catheter placements, but to a lesser extent than Gastroenterology. Score: 6/10.

4. **Research and Academic Opportunities**:
   - **Gastroenterology**: Rich in research opportunities, especially in areas like inflammatory bowel disease, hepatology, and advanced endoscopic techniques. Score: 8/10.
   - **Nephrology**: Offers substantial opportunities in both clinical and basic research, particularly in areas like dialysis, transplant, and hypertension. Score: 7/10.
5. **Patient Relationships and Continuity of Care**:
   - **Gastroenterology**: Offers a mix of acute and chronic patient care, with opportunities for long-term relationships in chronic GI and liver diseases. Score: 7/10.
   - **Nephrology**: Allows for long-term patient relationships, especially in chronic kidney disease management and dialysis. Score: 8/10.
6. **Career Opportunities and Demand**:
   - **Gastroenterology**: High demand, especially with the growing awareness of GI and liver diseases and the need for cancer screening. Score: 8/10.
   - **Nephrology**: Steady demand, particularly given the aging population and prevalence of conditions like hypertension and diabetes. Score: 7/10.
7. **Financial Compensation**:
   - **Gastroenterology**: Typically offers higher compensation due to procedural work and high demand. Score: 8/10.
   - **Nephrology**: Generally lower compared to some other specialties, but still offers a comfortable living. Score: 6/10.

Conclusion: Based on this analysis, I would recommend choosing a **Gastroenterology** fellowship. It scores higher in intellectual challenge, procedural involvement, research opportunities, career demand, and financial compensation. This choice should align with personal interests and career goals, especially if one is drawn to a diverse, procedure-intensive field with a mix of acute and chronic patient care. If a preference lies in fluid and electrolyte management and renal pathophysiology, then Nephrology might be more suitable.

Nephrology vs Geriatric Medicine

You

If you need to choose Nephrology or Geriatric Medicine fellowship, which one do you choose, you can describe but at the end you need choose one; each aspect comparisons may choose scores of 1-10.

ChatGPT

In choosing between a Nephrology and a Geriatric Medicine fellowship, we will evaluate various aspects such as intellectual challenge, work-life balance, procedural involvement, research opportunities, patient relationships, career demand, and financial compensation. Each aspect will be scored from 1 to 10 for both specialties, culminating in a final recommendation.

1. **Intellectual Challenge and Complexity**:
   - **Nephrology**: Offers deep insight into complex fluid, electrolyte, and acid-base balance, requiring a strong understanding of physiology and pathophysiology. It's intellectually demanding and constantly evolving. Score: 8/10.
   - **Geriatric Medicine**: Involves comprehensive care for older adults, addressing multiple, often complex health issues and polypharmacy. It requires a nuanced understanding of aging physiology and geriatric syndromes. Score: 8/10.
2. **Work-Life Balance**:
   - **Nephrology**: Generally offers a more predictable schedule compared to some other specialties, with fewer night shifts. It allows for a better work-life balance in most settings. Score: 8/10.
   - **Geriatric Medicine**: Often provides a favorable work-life balance, with fewer emergencies and a focus on outpatient or long-term care settings. Score: 9/10.
3. **Procedural Involvement**:
   - **Nephrology**: Involves procedures like kidney biopsies and dialysis catheter placements, but to a lesser extent than some other specialties. Score: 6/10.
   - **Geriatric Medicine**: Less procedural, focusing more on comprehensive assessment and management rather than invasive procedures. Score: 4/10.

4. **Research and Academic Opportunities**:
   - **Nephrology**: Offers substantial opportunities in both clinical and basic research, particularly in areas like dialysis, transplant, and hypertension. Score: 7/10.
   - **Geriatric Medicine**: Provides opportunities in research areas like aging, dementia, frailty, and polypharmacy, which are increasingly important in an aging society. Score: 7/10.
5. **Patient Relationships and Continuity of Care**:
   - **Nephrology**: Allows for long-term patient relationships, especially in chronic kidney disease management and dialysis. Score: 8/10.
   - **Geriatric Medicine**: Emphasizes long-term, holistic care for elderly patients, often building deep and lasting patient relationships. Score: 9/10.
6. **Career Opportunities and Demand**:
   - **Nephrology**: Steady demand, particularly given the aging population and prevalence of conditions like hypertension and diabetes. Score: 7/10.
   - **Geriatric Medicine**: Increasingly high demand due to the rapidly aging population and the unique healthcare needs of this group. Score: 9/10.
7. **Financial Compensation**:
   - **Nephrology**: Generally lower compared to some other specialties, but still offers a comfortable living. Score: 6/10.
   - **Geriatric Medicine**: Often not among the highest-paying specialties, but provides a stable and rewarding career. Score: 6/1[illegible]

Conclusion: Based on this analysis, I would recommend choosing a **Geriatric Medicine** fellowship. It scores higher in work-life balance, patient relationships, and career demand, particularly relevant in the context of an aging population and the growing need for specialists in elderly care. This choice should align with personal interests and career goals, especially if one is drawn to holistic, long-term care of the elderly. If a preference lies in more technical aspects like fluid and electrolyte management and renal pathophysiology, then Nephrology might be more suitable.

Geriatric Medicine vs Nephrology

You

If you need to choose Geriatric Medicine or Nephrology fellowship, which one do you choose, you can describe but at the end you need choose one; each aspect comparisons may choose scores of 1-10.

ChatGPT

In deciding between a Geriatric Medicine and a Nephrology fellowship, we will assess various aspects such as intellectual challenge, work-life balance, procedural involvement, research opportunities, patient relationships, career demand, and financial compensation. Each aspect will be scored from 1 to 10 for both specialties, leading to a final recommendation.

1. **Intellectual Challenge and Complexity**:
   - **Geriatric Medicine**: Involves comprehensive care for older adults, addressing multiple, often complex health issues and polypharmacy. It requires a nuanced understanding of aging physiology and geriatric syndromes. Score: 8/10.
   - **Nephrology**: Offers deep insight into complex fluid, electrolyte, and acid-base balance, requiring a strong understanding of physiology and pathophysiology. It's intellectually demanding and constantly evolving. Score: 8/10.
2. **Work-Life Balance**:
   - **Geriatric Medicine**: Often provides a favorable work-life balance, with fewer emergencies and a focus on outpatient or long-term care settings. Score: 9/10.
   - **Nephrology**: Generally offers a more predictable schedule compared to some other specialties, with fewer night shifts. It allows for a better work-life balance in most settings. Score: 8/10.
3. **Procedural Involvement**:
   - **Geriatric Medicine**: Less procedural, focusing more on comprehensive assessment and management rather than invasive procedures. Score: 4/10.
   - **Nephrology**: Involves procedures like kidney biopsies and dialysis catheter placements, but to a lesser extent than some other specialties. Score: 6/10.

4. **Research and Academic Opportunities**:
   - **Geriatric Medicine**: Provides opportunities in research areas like aging, dementia, frailty, and polypharmacy, which are increasingly important in an aging society. Score: 7/10.
   - **Nephrology**: Offers substantial opportunities in both clinical and basic research, particularly in areas like dialysis, transplant, and hypertension. Score: 7/10.
5. **Patient Relationships and Continuity of Care**:
   - **Geriatric Medicine**: Emphasizes long-term, holistic care for elderly patients, often building deep and lasting patient relationships. Score: 9/10.
   - **Nephrology**: Allows for long-term patient relationships, especially in chronic kidney disease management and dialysis. Score: 8/10.
6. **Career Opportunities and Demand**:
   - **Geriatric Medicine**: Increasingly high demand due to the rapidly aging population and the unique healthcare needs of this group. Score: 9/10.
   - **Nephrology**: Steady demand, particularly given the aging population and prevalence of conditions like hypertension and diabetes. Score: 7/10.
7. **Financial Compensation**:
   - **Geriatric Medicine**: Often not among the highest-paying specialties, but provides a stable and rewarding career. Score: 6/10.
   - **Nephrology**: Generally lower compared to some other specialties, but still offers a comfortable living. Score: 6/10.

Conclusion: Based on this analysis, I would recommend choosing a **Geriatric Medicine** fellowship. It scores higher in work-life balance, patient relationships, and career demand, particularly relevant in the context of an aging population and the growing need for specialists in elderly care. This choice should align with personal interests and career goals, especially if one is drawn to holistic, long-term care of the elderly. If a preference lies in more technical aspects like fluid and electrolyte management and renal pathophysiology, then Nephrology might be more suitable.

Nephrology vs Hematology and Oncology

You

If you need to choose Nephrology or Hematology and Oncology fellowship, which one do you choose, you can describe but at the end you need choose one; each aspect comparisons may choose scores of 1-10.

ChatGPT

In choosing between a Nephrology and a Hematology and Oncology fellowship, we will evaluate various aspects such as intellectual challenge, work-life balance, procedural involvement, research opportunities, patient relationships, career demand, and financial compensation. Each aspect will be scored from 1 to 10 for both specialties, culminating in a final recommendation.

1. **Intellectual Challenge and Complexity**:
   - **Nephrology**: Offers deep insight into complex fluid, electrolyte, and acid-base balance, requiring a strong understanding of physiology and pathophysiology. It's intellectually demanding and constantly evolving. Score: 8/10.
   - **Hematology and Oncology**: Involves a broad spectrum of diseases, including cancers and blood disorders. It requires a deep understanding of complex pathologies and rapidly evolving treatment modalities. Score: 9/10.
2. **Work-Life Balance**:
   - **Nephrology**: Generally offers a more predictable schedule compared to some other specialties, with fewer night shifts. It allows for a better work-life balance in most settings. Score: 8/10.
   - **Hematology and Oncology**: Can have variable schedules with on-call duties and the emotional demands of oncology care, which might affect work-life balance. Score: 6/10.
3. **Procedural Involvement**:
   - **Nephrology**: Involves procedures like kidney biopsies and dialysis catheter placements, but to a lesser extent than some other specialties. Score: 6/10.
   - **Hematology and Oncology**: Involves procedures such as bone marrow biopsies and administration of chemotherapy. Score: 7/10.

4. **Research and Academic Opportunities**:
   - **Nephrology**: Offers substantial opportunities in both clinical and basic research, particularly in areas like dialysis, transplant, and hypertension. Score: 7/10.
   - **Hematology and Oncology**: Rich in research opportunities, especially with the rapid advancements in cancer therapies and hematological treatments. Score: 9/10.
5. **Patient Relationships and Continuity of Care**:
   - **Nephrology**: Allows for long-term patient relationships, especially in chronic kidney disease management and dialysis. Score: 8/10.
   - **Hematology and Oncology**: Often involves intense, long-term relationships with patients undergoing cancer treatment or managing chronic blood disorders. Score: 8/10.
6. **Career Opportunities and Demand**:
   - **Nephrology**: Steady demand, particularly given the aging population and prevalence of conditions like hypertension and diabetes. Score: 7/10.
   - **Hematology and Oncology**: High demand, driven by the increasing prevalence of cancer and blood disorders. Score: 8/10.
7. **Financial Compensation**:
   - **Nephrology**: Generally lower compared to some other specialties, but still offers a comfortable living. Score: 6/10.
   - **Hematology and Oncology**: Typically offers higher compensation due to the complexity and intensity of the special[illegible]core: 8/10.

Conclusion: Based on this analysis, I would recommend choosing a **Hematology and Oncology** fellowship. It scores higher in intellectual challenge, research opportunities, career demand, and financial compensation. This choice should align with personal interests and career goals, especially if one is drawn to the dynamic and impactful field of cancer and blood disorders. If a preference lies in fluid and electrolyte management and renal pathophysiology, then Nephrology might be more suitable.

Hematology and Oncology vs Nephrology

You

If you need to choose Hematology and Oncology or Nephrology fellowship, which one do you choose, you can describe but at the end you need choose one; each aspect comparisons may choose scores of 1-10.

ChatGPT

In deciding between a Hematology and Oncology and a Nephrology fellowship, we will assess various aspects such as intellectual challenge, work-life balance, procedural involvement, research opportunities, patient relationships, career demand, and financial compensation. Each aspect will be scored from 1 to 10 for both specialties, leading to a final recommendation.

1. **Intellectual Challenge and Complexity**:
   - **Hematology and Oncology**: Involves a broad spectrum of diseases, including cancers and blood disorders. It requires a deep understanding of complex pathologies and rapidly evolving treatment modalities. Score: 9/10.
   - **Nephrology**: Offers deep insight into complex fluid, electrolyte, and acid-base balance, requiring a strong understanding of physiology and pathophysiology. It's intellectually demanding and constantly evolving. Score: 8/10.
2. **Work-Life Balance**:
   - **Hematology and Oncology**: Can have variable schedules with on-call duties and the emotional demands of oncology care, which might affect work-life balance. Score: 6/10.
   - **Nephrology**: Generally offers a more predictable schedule compared to some other specialties, with fewer night shifts. It allows for a better work-life balance in most settings. Score: 8/10.
3. **Procedural Involvement**:
   - **Hematology and Oncology**: Involves procedures such as bone marrow biopsies and administration of chemotherapy. Score: 7/10.
   - **Nephrology**: Involves procedures like kidney biopsies and dialysis catheter placements, but to a lesser extent than Hematology and Oncology. Score: 6/10.

4. **Research and Academic Opportunities**:
    - **Hematology and Oncology**: Rich in research opportunities, especially with the rapid advancements in cancer therapies and hematological treatments. Score: 9/10.
    - **Nephrology**: Offers substantial opportunities in both clinical and basic research, particularly in areas like dialysis, transplant, and hypertension. Score: 7/10.
5. **Patient Relationships and Continuity of Care**:
    - **Hematology and Oncology**: Often involves intense, long-term relationships with patients undergoing cancer treatment or managing chronic blood disorders. Score: 8/10.
    - **Nephrology**: Allows for long-term patient relationships, especially in chronic kidney disease management and dialysis. Score: 8/10.
6. **Career Opportunities and Demand**:
    - **Hematology and Oncology**: High demand, driven by the increasing prevalence of cancer and blood disorders. Score: 8/10.
    - **Nephrology**: Steady demand, particularly given the aging population and prevalence of conditions like hypertension and diabetes. Score: 7/10.
7. **Financial Compensation**:
    - **Hematology and Oncology**: Typically offers higher compensation due to the complexity and intensity of the specialty. Score: 8/10.
    - **Nephrology**: Generally lower compare[illegible] some other specialties, but still offers a comfortable living. Score: 6/10.

Conclusion: Based on this analysis, I would recommend choosing a **Hematology and Oncology** fellowship. It scores higher in intellectual challenge, research opportunities, career demand, and financial compensation. This choice should align with personal interests and career goals, especially if one is drawn to the dynamic and impactful field of cancer and blood disorders. If a preference lies in fluid and electrolyte management and renal pathophysiology, then Nephrology might be more suitable.

Nephrology vs Hospice and Palliative Medicine

You

If you need to choose Nephrology or Hospice and Palliative Medicine fellowship, which one do you choose, you can describe but at the end you need choose one; each aspect comparisons may choose scores of 1-10.

ChatGPT

In choosing between a Nephrology and a Hospice and Palliative Medicine fellowship, we will evaluate various aspects such as intellectual challenge, work-life balance, procedural involvement, research opportunities, patient relationships, career demand, and financial compensation. Each aspect will be scored from 1 to 10 for both specialties, culminating in a final recommendation.

1. **Intellectual Challenge and Complexity**:
   - **Nephrology**: Offers deep insight into complex fluid, electrolyte, and acid-base balance, requiring a strong understanding of physiology and pathophysiology. It's intellectually demanding and constantly evolving. Score: 8/10.
   - **Hospice and Palliative Medicine**: Involves comprehensive care focusing on symptom management, quality of life, and psychosocial aspects of patient care. It requires a nuanced understanding of a wide range of medical conditions and their impact on patients' lives. Score: 7/10.
2. **Work-Life Balance**:
   - **Nephrology**: Generally offers a more predictable schedule compared to some other specialties, with fewer night shifts. It allows for a better work-life balance in most settings. Score: 8/10.
   - **Hospice and Palliative Medicine**: Often provides a favorable work-life balance, with fewer emergencies and a focus on patient-centered care. Score: 9/10.
3. **Procedural Involvement**:
   - **Nephrology**: Involves procedures like kidney biopsies and dialysis catheter placements, but to a lesser extent than some other specialties. Score: 6/10.
   - **Hospice and Palliative Medicine**: Less procedural, focusing more on patient assessment, communication, and symptom management rather than invasive procedures. Score: 4/10.

4. **Research and Academic Opportunities**:
   - **Nephrology**: Offers substantial opportunities in both clinical and basic research, particularly in areas like dialysis, transplant, and hypertension. Score: 7/10.
   - **Hospice and Palliative Medicine**: Provides opportunities in research areas like symptom management, end-of-life care, and interdisciplinary care models. Score: 6/10.
5. **Patient Relationships and Continuity of Care**:
   - **Nephrology**: Allows for long-term patient relationships, especially in chronic kidney disease management and dialysis. Score: 8/10.
   - **Hospice and Palliative Medicine**: Emphasizes deep, meaningful patient relationships, often in the context of end-of-life care. Score: 9/10.
6. **Career Opportunities and Demand**:
   - **Nephrology**: Steady demand, particularly given the aging population and prevalence of conditions like hypertension and diabetes. Score: 7/10.
   - **Hospice and Palliative Medicine**: Increasing demand due to the aging population and a growing recognition of the importance of quality end-of-life care. Score: 8/10.
7. **Financial Compensation**:
   - **Nephrology**: Generally lower compared to some other specialties, but still offers a comfortable living. Score: 6/10.
   - **Hospice and Palliative Medicine**: Often not among the highest-paying specialties, but provides a rewarding and fulfilling career. Score: 6/10.

Conclusion: Based on this analysis, I would recommend choosing a **Hospice and Palliative Medicine** fellowship. It scores higher in work-life balance, patient relationships, and career demand, particularly relevant in the context of an aging population and the increasing need for quality end-of-life care. This choice should align with personal interests and career goals, especially if one is drawn to patient-centered, holistic care. If a preference lies in more technical aspects like fluid and electrolyte management and renal pathophysiology, then Nephrology might be more suitable.

Hospice and Palliative Medicine vs Nephrology

You

If you need to choose Hospice and Palliative Medicine or Nephrology fellowship, which one do you choose, you can describe but at the end you need choose one; each aspect comparisons may choose scores of 1-10.

ChatGPT

In deciding between a Hospice and Palliative Medicine and a Nephrology fellowship, we will assess various aspects such as intellectual challenge, work-life balance, procedural involvement, research opportunities, patient relationships, career demand, and financial compensation. Each aspect will be scored from 1 to 10 for both specialties, leading to a final recommendation.

1. **Intellectual Challenge and Complexity**:
   - **Hospice and Palliative Medicine**: Involves comprehensive care focusing on symptom management, quality of life, and psychosocial aspects of patient care. It requires a nuanced understanding of a wide range of medical conditions and their impact on patients' lives. Score: 7/10.
   - **Nephrology**: Offers deep insight into complex fluid, electrolyte, and acid-base balance, requiring a strong understanding of physiology and pathophysiology. It's intellectually demanding and constantly evolving. Score: 8/10.
2. **Work-Life Balance**:
   - **Hospice and Palliative Medicine**: Often provides a favorable work-life balance, with fewer emergencies and a focus on patient-centered care. Score: 9/10.
   - **Nephrology**: Generally offers a more predictable schedule compared to some other specialties, with fewer night shifts. It allows for a better work-life balance in most settings. Score: 8/10.
3. **Procedural Involvement**:
   - **Hospice and Palliative Medicine**: Less procedural, focusing more on patient assessment, communication, and symptom management rather than invasive procedures. Score: 4/10.
   - **Nephrology**: Involves procedures like kidney biopsies and dialysis catheter placements, but to a lesser extent than some other specialties. Score: 6/10.

4. **Research and Academic Opportunities**:
   - **Hospice and Palliative Medicine**: Provides opportunities in research areas like symptom management, end-of-life care, and interdisciplinary care models. Score: 6/10.
   - **Nephrology**: Offers substantial opportunities in both clinical and basic research, particularly in areas like dialysis, transplant, and hypertension. Score: 7/10.
5. **Patient Relationships and Continuity of Care**:
   - **Hospice and Palliative Medicine**: Emphasizes deep, meaningful patient relationships, often in the context of end-of-life care. Score: 9/10.
   - **Nephrology**: Allows for long-term patient relationships, especially in chronic kidney disease management and dialysis. Score: 8/10.
6. **Career Opportunities and Demand**:
   - **Hospice and Palliative Medicine**: Increasing demand due to the aging population and a growing recognition of the importance of quality end-of-life care. Score: 8/10.
   - **Nephrology**: Steady demand, particularly given the aging population and prevalence of conditions like hypertension and diabetes. Score: 7/10.
7. **Financial Compensation**:
   - **Hospice and Palliative Medicine**: Often not among the highest-paying specialties, but provides a rewarding and fulfilling career. Score: 6/10.
   - **Nephrology**: Generally lower compared to some other specialties, but still offers a comfortable living. Score: 6/10.

Conclusion: Based on this analysis, I would recommend choosing a **Hospice and Palliative Medicine** fellowship. It scores higher in work-life balance, patient relationships, and career demand, particularly relevant in the context of an aging population and the increasing need for quality end-of-life care. This choice should align with personal interests and career goals, especially if one is drawn to patient-centered, holistic care. If a preference lies in more technical aspects like fluid and electrolyte management and renal pathophysiology, then Nephrology might be more suitable.

Nephrology vs Oncology

You

If you need to choose Nephrology or Oncology fellowship, which one do you choose, you can describe but at the end you need choose one; each aspect comparisons may choose scores of 1-10.

ChatGPT

In choosing between a Nephrology and an Oncology fellowship, we will evaluate various aspects such as intellectual challenge, work-life balance, procedural involvement, research opportunities, patient relationships, career demand, and financial compensation. Each aspect will be scored from 1 to 10 for both specialties, culminating in a final recommendation.

1. **Intellectual Challenge and Complexity**:
   - **Nephrology**: Offers deep insight into complex fluid, electrolyte, and acid-base balance, requiring a strong understanding of physiology and pathophysiology. It's intellectually demanding and constantly evolving. Score: 8/10.
   - **Oncology**: Involves a broad spectrum of cancers, requiring a deep understanding of complex pathologies, evolving treatment modalities, and clinical trials. It's intellectually challenging and rapidly advancing. Score: 9/10.
2. **Work-Life Balance**:
   - **Nephrology**: Generally offers a more predictable schedule compared to Oncology, with fewer night shifts. It allows for a better work-life balance in most settings. Score: 8/10.
   - **Oncology**: Can have variable schedules with on-call duties and the emotional demands of cancer care, which might affect work-life balance. Score: 6/10.
3. **Procedural Involvement**:
   - **Nephrology**: Involves procedures like kidney biopsies and dialysis catheter placements, but to a lesser extent than some other specialties. Score: 6/10.
   - **Oncology**: Involves some procedures related to cancer diagnosis and treatment, such as bone marrow biopsies and administration of chemotherapy. Score: 7/10.

4. **Research and Academic Opportunities**:
   - **Nephrology**: Offers substantial opportunities in both clinical and basic research, particularly in areas like dialysis, transplant, and hypertension. Score: 7/10.
   - **Oncology**: Rich in research opportunities, especially with the rapid advancements in cancer therapies, personalized medicine, and immunotherapy. Score: 9/10.
5. **Patient Relationships and Continuity of Care**:
   - **Nephrology**: Allows for long-term patient relationships, especially in chronic kidney disease management and dialysis. Score: 8/10.
   - **Oncology**: Often involves intense, long-term relationships with patients undergoing cancer treatment. Score: 8/10.
6. **Career Opportunities and Demand**:
   - **Nephrology**: Steady demand, particularly given the aging population and prevalence of conditions like hypertension and diabetes. Score: 7/10.
   - **Oncology**: High demand, driven by the increasing prevalence of cancer and advancements in cancer treatment. Score: 8/10.
7. **Financial Compensation**:
   - **Nephrology**: Generally lower compared to some other specialties, but still offers a comfortable living. Score: 6/10.
   - **Oncology**: Typically offers higher compensation due to the complexity and intensity of the specialty. Score: 8/10.

Conclusion: Based on this analysis, I would recommend choosing an **Oncology** fellowship. It scores higher in intellectual challenge, research opportunities, career demand, and financial compensation. This choice should align with personal interests and career goals, especially if one is drawn to the dynamic and impactful field of cancer treatment and research. If a preference lies in fluid and electrolyte management and renal pathophysiology, then Nephrology might be more suitable.

Oncology vs Nephrology

You

If you need to choose Oncology or Nephrology fellowship, which one do you choose, you can describe but at the end you need choose one; each aspect comparisons may choose scores of 1-10.

ChatGPT

In deciding between an Oncology and a Nephrology fellowship, we will assess various aspects such as intellectual challenge, work-life balance, procedural involvement, research opportunities, patient relationships, career demand, and financial compensation. Each aspect will be scored from 1 to 10 for both specialties, leading to a final recommendation.

1. **Intellectual Challenge and Complexity**:
   - **Oncology**: Involves a broad spectrum of cancers, requiring a deep understanding of complex pathologies, evolving treatment modalities, and clinical trials. It's intellectually challenging and rapidly advancing. Score: 9/10.
   - **Nephrology**: Offers deep insight into complex fluid, electrolyte, and acid-base balance, requiring a strong understanding of physiology and pathophysiology. It's intellectually demanding and constantly evolving. Score: 8/10.
2. **Work-Life Balance**:
   - **Oncology**: Can have variable schedules with on-call duties and the emotional demands of cancer care, which might affect work-life balance. Score: 6/10.
   - **Nephrology**: Generally offers a more predictable schedule compared to Oncology, with fewer night shifts. It allows for a better work-life balance in most settings. Score: 8/10.

3. **Procedural Involvement**:
   - **Oncology**: Involves some procedures related to cancer diagnosis and treatment, such as bone marrow biopsies and administration of chemotherapy. Score: 7/10.
   - **Nephrology**: Involves procedures like kidney biopsies and dialysis catheter placements, but to a lesser extent than Oncology. Score: 6/10.
4. **Research and Academic Opportunities**:
   - **Oncology**: Rich in research opportunities, especially with the rapid advancements in cancer therapies, personalized medicine, and immunotherapy. Score: 9/10.
   - **Nephrology**: Offers substantial opportunities in both clinical and basic research, particularly in areas like dialysis, transplant, and hypertension. Score: 7/10.
5. **Patient Relationships and Continuity of Care**:
   - **Oncology**: Often involves intense, long-term relationships with patients undergoing cancer treatment. Score: 8/10.
   - **Nephrology**: Allows for long-term patient relationships, especially in chronic kidney disease management and dialysis. Score: 8/10.
6. **Career Opportunities and Demand**:
   - **Oncology**: High demand, driven by the increasing prevalence of cancer and advancements in cancer treatment. Score: 8/10.
   - **Nephrology**: Steady demand, particularly given the aging population and prevalence of conditions like hypertension and diabetes. Score: 7/10.
7. **Financial Compensation**:
   - **Oncology**: Typically offers higher compensation due to the complexity and intensity of the specialty. Score: 8/10.
   - **Nephrology**: Generally lower compared to some other specialties, but still offers a comfortable living. Score: 6/10.

Conclusion: Based on this analysis, I would recommend choosing an **Oncology** fellowship. It scores higher in intellectual challenge, research opportunities, career demand, and financial compensation. This choice should align with personal interests and career goals, especially if one is drawn to the dynamic and impactful field of cancer treatment and research. If a preference lies in fluid and electrolyte management and renal pathophysiology, then Nephrology might be more suitable.

Nephrology vs Pulmonary Disease

You

If you need to choose Nephrology or Pulmonary Disease fellowship, which one do you choose, you can describe but at the end you need choose one; each aspect comparisons may choose scores of 1-10.

ChatGPT

In choosing between a Nephrology and a Pulmonary Disease fellowship, we will evaluate various aspects such as intellectual challenge, work-life balance, procedural involvement, research opportunities, patient relationships, career demand, and financial compensation. Each aspect will be scored from 1 to 10 for both specialties, culminating in a final recommendation.

1. **Intellectual Challenge and Complexity**:
   - **Nephrology**: Offers deep insight into complex fluid, electrolyte, and acid-base balance, requiring a strong understanding of physiology and pathophysiology. It's intellectually demanding and constantly evolving. Score: 8/10.
   - **Pulmonary Disease**: Involves a broad spectrum of respiratory conditions, requiring a deep understanding of pulmonary physiology and pathophysiology. It's intellectually challenging and diverse. Score: 8/10.
2. **Work-Life Balance**:
   - **Nephrology**: Generally offers a more predictable schedule compared to Pulmonary Disease, with fewer night shifts. It allows for a better work-life balance in most settings. Score: 8/10.
   - **Pulmonary Disease**: Can have variable schedules with on-call duties and the demands of managing acute respiratory conditions. Score: 7/10.
3. **Procedural Involvement**:
   - **Nephrology**: Involves procedures like kidney biopsies and dialysis catheter placements, but to a lesser extent than Pulmonary Disease. Score: 6/10.
   - **Pulmonary Disease**: Often involves procedural skills, including bronchoscopies and thoracentesis, among others. Score: 8/10.

4. **Research and Academic Opportunities**:
   - **Nephrology**: Offers substantial opportunities in both clinical and basic research, particularly in areas like dialysis, transplant, and hypertension. Score: 7/10.
   - **Pulmonary Disease**: Provides significant opportunities for research in areas like COPD, asthma, interstitial lung disease, and sleep medicine. Score: 8/10.
5. **Patient Relationships and Continuity of Care**:
   - **Nephrology**: Allows for long-term patient relationships, especially in chronic kidney disease management and dialysis. Score: 8/10.
   - **Pulmonary Disease**: Often involves both acute and chronic care, with opportunities for long-term relationships in managing chronic respiratory diseases. Score: 7/10.
6. **Career Opportunities and Demand**:
   - **Nephrology**: Steady demand, particularly given the aging population and prevalence of conditions like hypertension and diabetes. Score: 7/10.
   - **Pulmonary Disease**: High demand, especially with the increasing prevalence of respiratory conditions like COPD and asthma. Score: 8/10.
7. **Financial Compensation**:
   - **Nephrology**: Generally lower compared to some other specialties, but still offers a comfortable living. Score: 6/10.
   - **Pulmonary Disease**: Typically offers higher compensation due to the procedural nature of the specialty and high demand. Score: 7/10.

Conclusion: Based on this analysis, I would recommend choosing a **Pulmonary Disease** fellowship. It scores slightly higher in procedural involvement, research opportunities, career demand, and financial compensation. This choice should align with personal interests and career goals, especially if one is drawn to the diverse and dynamic field of respiratory medicine. If a preference lies in fluid and electrolyte management and renal pathophysiology, then Nephrology might be more suitable.

Pulmonary Disease vs Nephrology

You

If you need to choose Pulmonary Disease or Nephrology fellowship, which one do you choose, you can describe but at the end you need choose one; each aspect comparisons may choose scores of 1-10.

ChatGPT

In deciding between a Pulmonary Disease and a Nephrology fellowship, we will assess various aspects such as intellectual challenge, work-life balance, procedural involvement, research opportunities, patient relationships, career demand, and financial compensation. Each aspect will be scored from 1 to 10 for both specialties, leading to a final recommendation.

1. **Intellectual Challenge and Complexity**:
   - **Pulmonary Disease**: Involves a broad spectrum of respiratory conditions, requiring a deep understanding of pulmonary physiology and pathophysiology. It's intellectually challenging and diverse. Score: 8/10.
   - **Nephrology**: Offers deep insight into complex fluid, electrolyte, and acid-base balance, requiring a strong understanding of physiology and pathophysiology. It's intellectually demanding and constantly evolving. Score: 8/10.
2. **Work-Life Balance**:
   - **Pulmonary Disease**: Can have variable schedules with on-call duties and the demands of managing acute respiratory conditions. Score: 7/10.
   - **Nephrology**: Generally offers a more predictable schedule compared to Pulmonary Disease, with fewer night shifts. It allows for a better work-life balance in most settings. Score: 8/10.
3. **Procedural Involvement**:
   - **Pulmonary Disease**: Often involves procedural skills, including bronchoscopies and thoracentesis, among others. Score: 8/10.
   - **Nephrology**: Involves procedures like kidney biopsies and dialysis catheter placements, but to a lesser extent than Pulmonary Disease. Score: 6/10.

4. **Research and Academic Opportunities**:
   - **Pulmonary Disease**: Provides significant opportunities for research in areas like COPD, asthma, interstitial lung disease, and sleep medicine. Score: 8/10.
   - **Nephrology**: Offers substantial opportunities in both clinical and basic research, particularly in areas like dialysis, transplant, and hypertension. Score: 7/10.
5. **Patient Relationships and Continuity of Care**:
   - **Pulmonary Disease**: Often involves both acute and chronic care, with opportunities for long-term relationships in managing chronic respiratory diseases. Score: 7/10.
   - **Nephrology**: Allows for long-term patient relationships, especially in chronic kidney disease management and dialysis. Score: 8/10.
6. **Career Opportunities and Demand**:
   - **Pulmonary Disease**: High demand, especially with the increasing prevalence of respiratory conditions like COPD and asthma. Score: 8/10.
   - **Nephrology**: Steady demand, particularly given the aging population and prevalence of conditions like hypertension and diabetes. Score: 7/10.
7. **Financial Compensation**:
   - **Pulmonary Disease**: Typically offers higher compensation due to the procedural nature of the specialty and high demand. Score: 7/10.
   - **Nephrology**: Generally lower compared to some other specialties, but still offers a comfortable living. Score: 6/10.

Conclusion: Based on this analysis, I would recommend choosing a **Pulmonary Disease** fellowship. It scores slightly higher in procedural involvement, research opportunities, career demand, and financial compensation. This choice should align with personal interests and career goals, especially if one is drawn to the diverse and dynamic field of respiratory medicine. If a preference lies in fluid and electrolyte management and renal pathophysiology, then Nephrology might be more suitable.

Nephrology or "Pulmonary Disease and Critical Care Medicine"

You

If you need to choose Nephrology or "Pulmonary Disease and Critical Care Medicine" fellowship, which one do you choose, you can describe but at the end you need choose one; each aspect comparisons may choose scores of 1-10.

ChatGPT

In choosing between a Nephrology and a "Pulmonary Disease and Critical Care Medicine" fellowship, we will evaluate various aspects such as intellectual challenge, work-life balance, procedural involvement, research opportunities, patient relationships, career demand, and financial compensation. Each aspect will be scored from 1 to 10 for both specialties, culminating in a final recommendation.

1. **Intellectual Challenge and Complexity**:
   - **Nephrology**: Offers deep insight into complex fluid, electrolyte, and acid-base balance, requiring a strong understanding of physiology and pathophysiology. It's intellectually demanding and constantly evolving. Score: 8/10.
   - **Pulmonary Disease and Critical Care Medicine**: Involves a broad spectrum of respiratory conditions and critical care scenarios, requiring a deep understanding of pulmonary physiology, critical illness, and mechanical ventilation. It's highly intellectually challenging and diverse. Score: 9/10.
2. **Work-Life Balance**:
   - **Nephrology**: Generally offers a more predictable schedule compared to Pulmonary and Critical Care, with fewer night shifts. It allows for a better work-life balance in most settings. Score: 8/10.
   - **Pulmonary Disease and Critical Care Medicine**: Often involves irregular hours and on-call duties due to the nature of critical care, which might affect work-life balance. Score: 6/10.
3. **Procedural Involvement**:
   - **Nephrology**: Involves procedures like kidney biopsies and dialysis catheter placements, but to a lesser extent than Pulmonary and Critical Care. Score: 6/10.
   - **Pulmonary Disease and Critical Care Medicine**: Highly procedural, involving advanced techniques like bronchoscopy, thoracentesis, and management of critically ill patients. Score: 9/10.

4. **Research and Academic Opportunities**:
   - **Nephrology**: Offers substantial opportunities in both clinical and basic research, particularly in areas like dialysis, transplant, and hypertension. Score: 7/10.
   - **Pulmonary Disease and Critical Care Medicine**: Provides significant opportunities for research in areas like ARDS, COPD, asthma, and mechanical ventilation. Score: 8/10.
5. **Patient Relationships and Continuity of Care**:
   - **Nephrology**: Allows for long-term patient relationships, especially in chronic kidney disease management and dialysis. Score: 8/10.
   - **Pulmonary Disease and Critical Care Medicine**: Often involves both acute care in critical settings and long-term management of chronic pulmonary diseases. Score: 7/10.
6. **Career Opportunities and Demand**:
   - **Nephrology**: Steady demand, particularly given the aging population and prevalence of conditions like hypertension and diabetes. Score: 7/10.
   - **Pulmonary Disease and Critical Care Medicine**: High demand, especially with the increasing prevalence of respiratory co[illegible]itions and the critical care needs of an aging population. Score: 8/10.

7. **Financial Compensation**:
   - **Nephrology**: Generally lower compared to some other specialties, but still offers a comfortable living. Score: 6/10.
   - **Pulmonary Disease and Critical Care Medicine**: Typically offers higher compensation due to the specialized nature of the field and the intensity of critical care work. Score: 8/10.

Conclusion: Based on this analysis, I would recommend choosing a **Pulmonary Disease and Critical Care Medicine** fellowship. It scores higher in intellectual challenge, procedural involvement, research opportunities, career demand, and financial compensation. This choice should align with personal interests and career goals, especially if one is drawn to a dynamic, high-intensity field with a focus on respiratory and critical care medicine. If a preference lies in fluid and electrolyte management and renal pathophysiology, then Nephrology might be more suitable.

“Pulmonary Disease and Critical Care Medicine” vs Nephrology

You

If you need to choose “Pulmonary Disease and Critical Care Medicine” or Nephrology fellowship, which one do you choose, you can describe but at the end you need choose one; each aspect comparisons may choose scores of 1-10.

ChatGPT

In deciding between a "Pulmonary Disease and Critical Care Medicine" and a Nephrology fellowship, we will assess various aspects such as intellectual challenge, work-life balance, procedural involvement, research opportunities, patient relationships, career demand, and financial compensation. Each aspect will be scored from 1 to 10 for both specialties, leading to a final recommendation.

1. **Intellectual Challenge and Complexity**:
   - **Pulmonary Disease and Critical Care Medicine**: Involves a broad spectrum of respiratory conditions and critical care scenarios, requiring a deep understanding of pulmonary physiology, critical illness, and mechanical ventilation. It's highly intellectually challenging and diverse. Score: 9/10.
   - **Nephrology**: Offers deep insight into complex fluid, electrolyte, and acid-base balance, requiring a strong understanding of physiology and pathophysiology. It's intellectually demanding and constantly evolving. Score: 8/10.
2. **Work-Life Balance**:
   - **Pulmonary Disease and Critical Care Medicine**: Often involves irregular hours and on-call duties due to the nature of critical care, which might affect work-life balance. Score: 6/10.
   - **Nephrology**: Generally offers a more predictable schedule compared to Pulmonary and Critical Care, with fewer night shifts[illegible] allows for a better work-life balance in most settings. Score: 8/10.
3. **Procedural Involvement**:
   - **Pulmonary Disease and Critical Care Medicine**: Highly procedural, involving advanced techniques like bronchoscopy, thoracentesis, and management of critically ill patients. Score: 9/10.
   - **Nephrology**: Involves procedures like kidney biopsies and dialysis catheter placements, but to a lesser extent than Pulmonary and Critical Care. Score: 6/10.

4. **Research and Academic Opportunities**:
   - **Pulmonary Disease and Critical Care Medicine**: Provides significant opportunities for research in areas like ARDS, COPD, asthma, and mechanical ventilation. Score: 8/10.
   - **Nephrology**: Offers substantial opportunities in both clinical and basic research, particularly in areas like dialysis, transplant, and hypertension. Score: 7/10.
5. **Patient Relationships and Continuity of Care**:
   - **Pulmonary Disease and Critical Care Medicine**: Often involves both acute care in critical settings and long-term management of chronic pulmonary diseases. Score: 7/10.
   - **Nephrology**: Allows for long-term patient relationships, especially in chronic kidney disease management and dialysis. Score: 8/10.
6. **Career Opportunities and Demand**:
   - **Pulmonary Disease and Critical Care Medicine**: High demand, especially with the increasing prevalence of respiratory conditions and the critical care needs of an aging population. Score: 8/10.
   - **Nephrology**: Steady demand, particularly given the aging population and prevalence of conditions like hypertension and diabetes. Score: 7/10.

7. **Financial Compensation**:
   - **Pulmonary Disease and Critical Care Medicine**: Typically offers higher compensation due to the specialized nature of the field and the intensity of critical care work. Score: 8/10.
   - **Nephrology**: Generally lower compared to some other specialties, but still offers a comfortable living. Score: 6/10.

Conclusion: Based on this analysis, I would recommend choosing a **Pulmonary Disease and Critical Care Medicine** fellowship. It scores higher in intellectual challenge, procedural involvement, research opportunities, career demand, and financial compensation. This choice should align with personal interests and career goals, especially if one is drawn to a dynamic, high-intensity field with a focus on respiratory and critical care medicine. If a preference lies in fluid and electrolyte management and renal pathophysiology, then Nephrology might be more suitable.

Nephrology vs Rheumatology

You

If you need to choose Nephrology or Rheumatology fellowship, which one do you choose, you can describe but at the end you need choose one; each aspect comparisons may choose scores of 1-10.

ChatGPT

In choosing between a Nephrology and a Rheumatology fellowship, we will evaluate various aspects such as intellectual challenge, work-life balance, procedural involvement, research opportunities, patient relationships, career demand, and financial compensation. Each aspect will be scored from 1 to 10 for both specialties, culminating in a final recommendation.

1. **Intellectual Challenge and Complexity**:
   - **Nephrology**: Offers deep insight into complex fluid, electrolyte, and acid-base balance, requiring a strong understanding of physiology and pathophysiology. It's intellectually demanding and constantly evolving. Score: 8/10.
   - **Rheumatology**: Involves a broad spectrum of autoimmune and musculoskeletal diseases, requiring a deep understanding of immunology and systemic manifestations of these diseases. It's intellectually challenging and diverse. Score: 8/10.
2. **Work-Life Balance**:
   - **Nephrology**: Generally offers a more predictable schedule compared to some other specialties, with fewer night shifts. It allows for a better work-life balance in most settings. Score: 8/10.
   - **Rheumatology**: Also known for a favorable work-life balance, with fewer emergencies and a focus on outpatient care. Score: 9/10.
3. **Procedural Involvement**:
   - **Nephrology**: Involves procedures like kidney biopsies and dialysis catheter placements, but to a lesser extent than some other specialties. Score: 6/10.
   - **Rheumatology**: Involves procedures such as joint injections and aspirations, but overall less procedural than Nephrology. Score: 5/10.

4. **Research and Academic Opportunities**:
    - **Nephrology**: Offers substantial opportunities in both clinical and basic research, particularly in areas like dialysis, transplant, and hypertension. Score: 7/10.
    - **Rheumatology**: Provides significant opportunities for research in autoimmune diseases, biologic therapies, and novel immunomodulators. Score: 8/10.
5. **Patient Relationships and Continuity of Care**:
    - **Nephrology**: Allows for long-term patient relationships, especially in chronic kidney disease management and dialysis. Score: 8/10.
    - **Rheumatology**: Often involves long-term management of chronic diseases, building lasting patient relationships. Score: 8/10.
6. **Career Opportunities and Demand**:
    - **Nephrology**: Steady demand, particularly given the aging population and prevalence of conditions like hypertension and diabetes. Score: 7/10.
    - **Rheumatology**: High demand, especially with the increasing recognition of autoimmune diseases and advancements in treatments. Score: 8/10.

7. **Financial Compensation**:
    - **Nephrology**: Generally lower compared to some other specialties, but still offers a comfortable living. Score: 6/10.
    - **Rheumatology**: Comparable to Nephrology in terms of compensation, not among the highest-paying specialties but offers a stable income. Score: 6/10.

Conclusion: Based on this analysis, I would recommend choosing a **Rheumatology** fellowship. It scores slightly higher in work-life balance, research opportunities, and career demand, particularly relevant in the context of the growing need for specialists in autoimmune and musculoskeletal diseases. This choice should align with personal interests and career goals, especially if one is drawn to the field of immunology and long-term management of chronic diseases. If a preference lies in fluid and electrolyte management and renal pathophysiology, then Nephrology might be more suitable.

Rheumatology vs Nephrology

You

If you need to choose Rheumatology or Nephrology fellowship, which one do you choose, you can describe but at the end you need choose one; each aspect comparisons may choose scores of 1-10.

ChatGPT

In deciding between a Rheumatology and a Nephrology fellowship, we will assess various aspects such as intellectual challenge, work-life balance, procedural involvement, research opportunities, patient relationships, career demand, and financial compensation. Each aspect will be scored from 1 to 10 for both specialties, leading to a final recommendation.

1. **Intellectual Challenge and Complexity**:
   - **Rheumatology**: Involves a broad spectrum of autoimmune and musculoskeletal diseases, requiring a deep understanding of immunology and systemic manifestations of these diseases. It's intellectually challenging and diverse. Score: 8/10.
   - **Nephrology**: Offers deep insight into complex fluid, electrolyte, and acid-base balance, requiring a strong understanding of physiology and pathophysiology. It's intellectually demanding and constantly evolving. Score: 8/10.
2. **Work-Life Balance**:
   - **Rheumatology**: Known for a favorable work-life balance, with fewer emergencies and a focus on outpatient care. Score: 9/10.
   - **Nephrology**: Generally offers a more predictable schedule compared to some other specialties, with fewer night shifts. It allows for a better work-life balance in most settings. Score: 8/10.
3. **Procedural Involvement**:
   - **Rheumatology**: Involves procedures such as joint injections and aspirations, but overall less procedural than Nephrology. Score: 5/10.
   - **Nephrology**: Involves procedures like kidney biopsies and dialysis catheter placements, but to a lesser extent than some other specialties. Score: 6/10.

4. **Research and Academic Opportunities**:
   - **Rheumatology**: Provides significant opportunities for research in autoimmune diseases, biologic therapies, and novel immunomodulators. Score: 8/10.
   - **Nephrology**: Offers substantial opportunities in both clinical and basic research, particularly in areas like dialysis, transplant, and hypertension. Score: 7/10.
5. **Patient Relationships and Continuity of Care**:
   - **Rheumatology**: Often involves long-term management of chronic diseases, building lasting patient relationships. Score: 8/10.
   - **Nephrology**: Allows for long-term patient relationships, especially in chronic kidney disease management and dialysis. Score: 8/10.
6. **Career Opportunities and Demand**:
   - **Rheumatology**: High demand, especially with the increasing recognition of autoimmune diseases and advancements in treatments. Score: 8/10.
   - **Nephrology**: Steady demand, particularly given the aging population and prevalence of conditions like hypertension and diabetes. Score: 7/10.
7. **Financial Compensation**:
   - **Rheumatology**: Comparable to Nephrology in terms of compensation, not among the highest-paying specialties but offers a stable income. Score: 6/10.
   - **Nephrology**: Generally lower compared to some other specialties, but still offers a comfortable living. Score: 6/10.

Conclusion: Based on this analysis, I would recommend choosing a **Rheumatology** fellowship. It scores slightly higher in work-life balance, research opportunities, and career demand, particularly relevant in the context of the growing need for specialists in autoimmune and musculoskeletal diseases. This choice should align with personal interests and career goals, especially if one is drawn to the field of immunology and long-term management of chronic diseases. If a preference lies in fluid and electrolyte management and renal pathophysiology, then Nephrology might be more suitable.

Nephrology vs Sleep Medicine

You

If you need to choose Nephrology or Sleep Medicine fellowship, which one do you choose, you can describe but at the end you need choose one; each aspect comparisons may choose scores of 1-10.

ChatGPT

In choosing between a Nephrology and a Sleep Medicine fellowship, we will evaluate various aspects such as intellectual challenge, work-life balance, procedural involvement, research opportunities, patient relationships, career demand, and financial compensation. Each aspect will be scored from 1 to 10 for both specialties, culminating in a final recommendation.

1. **Intellectual Challenge and Complexity**:
   - **Nephrology**: Offers deep insight into complex fluid, electrolyte, and acid-base balance, requiring a strong understanding of physiology and pathophysiology. It's intellectually demanding and constantly evolving. Score: 8/10.
   - **Sleep Medicine**: Involves a broad understanding of sleep disorders, their pathophysiology, and the interplay with other medical conditions. It's intellectually stimulating and integrates knowledge from various fields. Score: 7/10.
2. **Work-Life Balance**:
   - **Nephrology**: Generally offers a more predictable schedule compared to some other specialties, with fewer night shifts. It allows for a better work-life balance in most settings. Score: 8/10.
   - **Sleep Medicine**: Known for a favorable [illegible]rk-life balance, often with regular hours and fewer emergencies. Score: 9/10.

3. **Procedural Involvement**:
   - **Nephrology**: Involves procedures like kidney biopsies and dialysis catheter placements, but to a lesser extent than some other specialties. Score: 6/10.
   - **Sleep Medicine**: Primarily diagnostic rather than procedural, focusing on sleep studies and interpretation. Score: 4/10.
4. **Research and Academic Opportunities**:
   - **Nephrology**: Offers substantial opportunities in both clinical and basic research, particularly in areas like dialysis, transplant, and hypertension. Score: 7/10.
   - **Sleep Medicine**: Provides opportunities for research in sleep disorders, circadian rhythms, and their impact on overall health. Score: 6/10.
5. **Patient Relationships and Continuity of Care**:
   - **Nephrology**: Allows for long-term patient relationships, especially in chronic kidney disease management and dialysis. Score: 8/10.
   - **Sleep Medicine**: Often involves long-term management of sleep disorders, but the patient contact is typically less intensive than in Nephrology. Score: 7/10.
6. **Career Opportunities and Demand**:
   - **Nephrology**: Steady demand, particularly given the aging population and prevalence of conditions like hypertension and diabetes. Score: 7/10.
   - **Sleep Medicine**: Growing demand due to increased recognition of the importance of sleep health, but it's a smaller field compared to Nephrology. Score: 6/10.
7. **Financial Compensation**:
   - **Nephrology**: Generally lower compared to some other specialties, but still offers a comfortable living. Score: 6/10.
   - **Sleep Medicine**: Compensation can vary, often depending on the practice setting and additional board certifications. Score: 6/10.

Conclusion: Based on this analysis, I would recommend choosing a **Nephrology** fellowship. While Sleep Medicine offers a better work-life balance and is less procedurally intensive, Nephrology scores higher in intellectual challenge, research opportunities, and career demand. This choice should align with personal interests and career goals, especially if one is drawn to complex pathophysiological processes and long-term patient care. If a preference lies in a more lifestyle-friendly specialty with a focus on sleep-related disorders, then Sleep Medicine might be more suitable.

Sleep Medicine vs Nephrology

You

If you need to choose Sleep Medicine or Nephrology fellowship, which one do you choose, you can describe but at the end you need choose one; each aspect comparisons may choose scores of 1-10.

ChatGPT

In deciding between a Sleep Medicine and a Nephrology fellowship, we will assess various aspects such as intellectual challenge, work-life balance, procedural involvement, research opportunities, patient relationships, career demand, and financial compensation. Each aspect will be scored from 1 to 10 for both specialties, leading to a final recommendation.

1. **Intellectual Challenge and Complexity**:
   - **Sleep Medicine**: Involves a broad understanding of sleep disorders, their pathophysiology, and the interplay with other medical conditions. It's intellectually stimulating and integrates knowledge from various fields. Score: 7/10.
   - **Nephrology**: Offers deep insight into complex fluid, electrolyte, and acid-base balance, requiring a strong understanding of physiology and pathophysiology. It's intellectually demanding and constantly evolving. Score: 8/10.
2. **Work-Life Balance**:
   - **Sleep Medicine**: Known for a favorable work-life balance, often with regular hours and fewer emergencies. Score: 9/10.
   - **Nephrology**: Generally offers a more predictable schedule compared to some other specialties, with fewer night shifts. It allows for a better work-life balance in most settings. Score: 8/10.
3. **Procedural Involvement**:
   - **Sleep Medicine**: Primarily diagnostic rather than procedural, focusing on sleep studies and interpretation. Score: 4/10.
   - **Nephrology**: Involves procedures like kidney biopsies and dialysis catheter placements, but to a lesser extent than some other specialties. Score: 6/10.

4. **Research and Academic Opportunities**:
   - **Sleep Medicine**: Provides opportunities for research in sleep disorders, circadian rhythms, and their impact on overall health. Score: 6/10.
   - **Nephrology**: Offers substantial opportunities in both clinical and basic research, particularly in areas like dialysis, transplant, and hypertension. Score: 7/10.
5. **Patient Relationships and Continuity of Care**:
   - **Sleep Medicine**: Often involves long-term management of sleep disorders, but the patient contact is typically less intensive than in Nephrology. Score: 7/10.
   - **Nephrology**: Allows for long-term patient relationships, especially in chronic kidney disease management and dialysis. Score: 8/10.
6. **Career Opportunities and Demand**:
   - **Sleep Medicine**: Growing demand due to increased recognition of the importance of sleep health, but it's a smaller field compared to Nephrology. Score: 6/10.
   - **Nephrology**: Steady demand, particularly given the aging population and prevalence of conditions like hypertension and diabetes. Score: 7/10.
7. **Financial Compensation**:
   - **Sleep Medicine**: Compensation can vary, often depending on the practice setting and additional board certifications. Score: 6/10.
   - **Nephrology**: Generally lower compared to some other specialties, but still offers a comfortable living. Score: 6/10.

Conclusion: Based on this analysis, I would recommend choosing a **Nephrology** fellowship. While Sleep Medicine offers a better work-life balance and is less procedurally intensive, Nephrology scores higher in intellectual challenge, research opportunities, and career demand. This choice should align with personal interests and career goals, especially if one is drawn to complex pathophysiological processes and long-term patient care. If a preference lies in a more lifestyle-friendly specialty with a focus on sleep-related disorders, then Sleep Medicine might be more suitable.
